# Amino Acid Properties Conserved in Molecular Evolution

**DOI:** 10.1371/journal.pone.0098983

**Published:** 2014-06-26

**Authors:** Witold R. Rudnicki, Teresa Mroczek, Paweł Cudek

**Affiliations:** 1 Interdisciplinary Centre for Mathematical and Computational Modelling, Warsaw University, Warsaw, Poland; 2 Chair of Expert Systems and Artificial Intelligence, University of Information Technology and Management, Rzeszow, Poland; The Centre for Research and Technology, Hellas, Greece

## Abstract

That amino acid properties are responsible for the way protein molecules evolve is natural and is also reasonably well supported both by the structure of the genetic code and, to a large extent, by the experimental measures of the amino acid similarity. Nevertheless, there remains a significant gap between observed similarity matrices and their reconstructions from amino acid properties. Therefore, we introduce a simple theoretical model of amino acid similarity matrices, which allows splitting the matrix into two parts – one that depends only on mutabilities of amino acids and another that depends on pairwise similarities between them. Then the new synthetic amino acid properties are derived from the pairwise similarities and used to reconstruct similarity matrices covering a wide range of information entropies. Our model allows us to explain up to 94% of the variability in the BLOSUM family of the amino acids similarity matrices in terms of amino acid properties. The new properties derived from amino acid similarity matrices correlate highly with properties known to be important for molecular evolution such as hydrophobicity, size, shape and charge of amino acids. This result closes the gap in our understanding of the influence of amino acids on evolution at the molecular level. The methods were applied to the single family of similarity matrices used often in general sequence homology searches, but it is general and can be used also for more specific matrices. The new synthetic properties can be used in analyzes of protein sequences in various biological applications.

## Introduction

The connection between amino acid properties and molecular evolution was proposed very soon after the discovery of the latter [1]–[4]. It has been shown that the most frequently occurring single nucleotide mutations of DNA lead to amino acid changes that conserve certain amino acid properties [3], [5]. It has also been suggested that this property of the genetic code was acquired in the process of evolution – the code itself evolved to minimize changes in important properties of amino acids upon mutation [5]–[10]. Those mutations which conserve important residue properties are much more likely to preserve the structure and function of protein, than those which dramatically change these properties. Therefore, the importance of the amino acid properties should be reflected in the data on the actual mutations of proteins. Such data can be collected in the laboratory by studying the effects of mutations on protein activity in numerous mutants of various proteins. These mutants can be obtained via site-directed mutagenesis, and one can measure directly how mutations affect protein activity [11], [12]. However, the amount of work necessary to perform such analysis limits practical application of this approach, since only a small subset of all possible combinations of mutations can be studied in this way. One can also study the mutational experiments performed by Nature, using proteins from all living organisms as experimental material. The evolving proteins retain their structure and function, so by studying the mutations occurring in natural sequences, and observing which properties are conserved in these mutations, one may find which properties are indeed important. Compact and synthetic information on the mutations occurring in nature is available in the form of amino acid similarity matrices (AASMs). Originally introduced by Dayhoff and Eck in 1968 [13], the AASMs were subsequently developed by several researchers. These matrices are used for measuring the similarity of proteins by algorithms such as Smith-Waterman [14] or BLAST [15]. Currently, the most often used are of two types: PAM [16], based on the original approach of Dayhoff and Eck, and BLOSUM [17] introduced in 1992, which is based on a slightly different method. It has been shown that AASMs correlate quite strongly with the amino acid properties [18]. It has also been shown that mutation matrices specific to given protein families can exhibit even stronger correlations [19]. These findings are good evidence supporting the role amino acid properties play in molecular evolution.

Nevertheless, while the fact that the properties of amino acids are decisive in determining protein structure, function and evolution on the molecular level is now universally accepted, and even considered self-evident, the support for the last thesis is not yet satisfactory. The structure of the genetic code might have arisen as a result of an evolutionary process that minimized errors introduced by single-step non-synonymous codon mutations, but this is still disputed [20] and alternative theories for the explanation of the genetic code structure are being investigated [21]–[25]. Also, only up to 85% of the variability within AASMs is explained by the model which relates distance matrices created from the differences in chemical properties of amino acids to similarity matrices [18], leaving the remainder unexplained. As early as 1965 Zuckerkandl and Pauling [1], stated “apparently chemists and protein molecules do not share the same opinions regarding the definition of the most prominent properties of a residue”. This remains true to a large extent also today, despite the hundreds of various amino acid descriptors proposed up to date.

In the current paper we propose to apply the inverse engineering approach to find the connections between evolutionary and chemical properties of amino acids. In this approach first we build the simple model of amino acids evolution that allows us to represent AASM as a sum of two matrices – one that depends only on global properties of amino acids (mutabilities), and the other that depends on similarity between amino acids. Then we derive fundamental vectors describing the second matrix and compare them with the amino acid properties. We also analyse similarities between these fundamental vectors obtained from all matrices in the series of BLOSUM matrices. Finally we reconstruct entire series of BLOSUM matrices with high fidelity using single set of fundamental vectors.

## Materials and Methods

### Amino acid similarity matrices

The AASM is a compact representation of molecular evolution. It has been shown by Altschul [26], that all AASM can be represented in the following way:
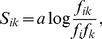
(1)where *S_ik_* is the matrix element *f_i_* and *f_k_* are the background frequencies of the *i-th* and *k-th* amino acid and *f_ik_* is the frequency of exchange between them. Generally it is impossible to tell the direction of mutations, hence it is assumed that observed frequencies are the sum of mutations in both directions. It follows that



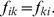
(2)One should note that the diagonal and off-diagonal elements of the matrices represent different types of information. The diagonal terms correspond to the observed levels of conservation of amino acids. Thus the diagonal term is a property of a single amino acid that measures how similar it is on average to all other amino acids. The off-diagonal terms are on the other hand related with the similarity between amino acids. Nevertheless, one should note that the differences between mutabilities of amino acids are also reflected in the off-diagonal terms.

The most often used similarity matrices are those that are offered as choices in the BLAST service from NCBI. They belong either to the PAM (PAM30, PAM70 and PAM250) or BLOSUM (BLOSUM90, BLOSUM80, BLOSUM62, BLOSUM50 and BLOSUM45) family of matrices. The PAM30 and PAM70 matrices are suitable mostly for searches of very short sequences with high similarity, whereas PAM250 is present mostly for historical reasons – it has been the most often used matrix before introduction of modern BLOSUM series.

PAM matrices are obtained as an extrapolation from the mutations observed in the closely related proteins, whereas BLOSUM matrices were derived from direct observation of mutations in conserved sequence blocks in proteins of variable similarity. The exchange frequencies *f_ik_* in BLOSUM matrices are obtained from direct observation, whereas in the PAM matrices the more elaborate procedure was applied for their derivation. Nevertheless, it can be shown that the final result of PAM procedure can be written in form of Eq. 1.

The BLOSUM matrices are generally considered superior to PAM matrices and are recommended for BLAST searches. BLOSUM62 in particular is considered the best matrix for wide range of similarities and query lengths and is the default choice in BLAST, but can be replaced by BLOSUM80 for short queries and BLOSUM45 for searches of weakly similar sequences. The ordinal number attached to BLOSUM name is the level of clustering that was applied to the BLOCKS database before derivation of the BLOSUM matrix. All sequences that have a similarity higher than the **x**% are represented by a single averaged entry in the clustered database used for derivation of BLOSUM**x** matrix.

Therefore the analysis performed in the current study was limited to the BLOSUM family of matrices. The analysis covered the entire range of matrices from BLOSUM100 to BLOSUM30, with step 5, and additionally the BLOSUM62 matrix. The matrices that are most often used in similarity searches are scaled and rounded to the nearest integer, however, the rounding procedure introduces an unnecessary source of error when correlations with properties are studied. Therefore the non-rounded versions of the matrices were used in the current study.

The notions of mutability and relative mutability introduced for derivation of PAM matrices are useful for the current study. The **mutability** of amino acid *k* is defined here as:
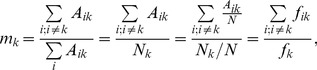
(3)where *A_ik_* is the number of counts of *i-th* and *k-th* amino acid in the same position within the conserved block of alignments; *N_k_* is the number of counts of *k-th* amino acid in the data set and N is the total number of counts of all amino acids in the data set. The **relative mutability** was defined by Dayhoff et al arbitrarily in relation to the mutability of alanine:



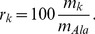
(4)Both mutability and relative mutability were defined in the context of the original data set used for derivation of PAM matrices. In the current study mutability the Eq. 3 is used to define mutability corresponding to any similarity matrix defined by Eq. 1. The relative mutability *r* is redefined with respect to the average mutability of all amino acids in this data set:
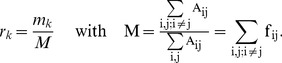
(5)


The Eq. 5 is more general than the original definition of Dayhof and co-workers.

Any similarity matrix defined by Eq. 1 can be transformed to the following form
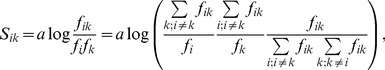
(6)


and taking into account Eq. 3 this can be rewritten as:
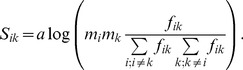
(7)


Therefore one can decompose the similarity matrix into two terms
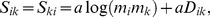
(8)


with *D_ik_* defined as:
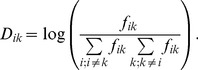
(9)


Both terms in the Eq. 7 depend on the evolutionary divergence of the underlying mutation matrix. One can remove this dependence from the first term by rewriting the Eq. 6 in terms of relative mutabilities, defined in the Eq. 4 in the following way:
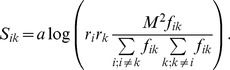
(10)


Consequently, the similarity matrix S can be expressed as
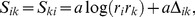
(11)


with 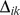 defined as
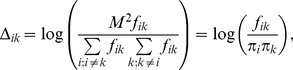
(12)where 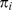 is defined as a fraction of mutations involving *i-th* amino acid in all mutations:



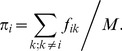
(13)One can notice that both *D_ik_* and 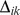 are proportional to the logarithm of odds of mutation frequencies. The first term in Eq. 8 and Eq. 11 does not depend on the specific pair-wise similarities between amino acids. The second term describes pair similarity between the *i-th* and *k-th* amino acid. It is not clear a priori, whether Eq. 8 or rather Eq. 11 is better suited for the purpose of our study, thus both equations were examined.

As demonstrated above, the original AASM can be decomposed into two parts. The first one is further referred to as G-matrix, since it is obtained in terms of amino acid mutabilities that are global properties of single amino acids. The second one describes pair-wise similarities and is further referred to as P-matrix. In matrix notation:
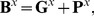
(14)where **B** ^x^ is the BLOSUMx matrix, **G** ^x^ is the G-matrix and **P** ^x^ is the P-matrix obtained from **B** ^x^. In the absence of preferences between any pair of amino acids the **G** matrix would still be non-uniform, unless the mutabilities of amino acids were all equal.

### Amino acid properties

It has previously been established that the polarity, hydrophobicity and size of amino acids are correlated with the AASMs [2], [4], [6], [8], [18]. Despite the numerous proposed descriptors for polarity, size and hydrophobicity, the right set for describing protein evolution has not been found. There is no fundamental reason why the properties that we can measure should be the most convenient ones for modelling the process of evolution.

In the current work it is assumed that the currently known descriptors may not be the most important for evolution. Instead we propose that synthetic properties that can be derived directly from the P-matrix are best suited to describe evolution on the molecular level. To this end the eigenvector decomposition of the P-matrix is used. The square 20×20 matrix **P** that has linearly independent columns can be represented in the following form:
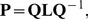
(15)where **Q** is the matrix of eigenvectors and **L** is a diagonal matrix of eigenvalues. Both **Q** and **L** are square 20×20 matrices. The *i-th* column of the Q matrix (and i-th row of the **Q** ^−1^ matrix) is the i-th eigenvector of **P**. The *i-th* element of the diagonal of matrix **L** is the *i-th* eigenvalue of **P**. Due to properties of matrix multiplication the compact formula in Eq. 15 is equivalent to the following sum:
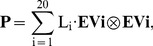
(16)where *L_i_* is the i-th eigenvalue, **EV**i is the *i-th* eigenvector and 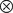 denotes outer product of vectors. Hence, the P-matrix is represented as a sum of 20 matrices, each constructed using single eigenvector and corresponding eigenvalue. The eigenvectors are ordered according to the absolute value of the corresponding eigenvalue, therefore the terms of sum in Eq. 16 are also ordered. Thus truncation of the series at *k*-th term gives approximation containing *k* most important contributions.

We postulate that eigenvectors of P-matrices corresponding to large eigenvalues are the synthetic properties that best describe evolution on molecular level.

If this postulate is correct then several consequences should follow:

1. Firstly, the eigenvectors of matrices in the entire series should be conserved – the eigenvectors derived from BLOSUM100 matrix should be similar to those derived from BLOSUM45 and even BLOSUM30 matrix.
2. Secondly, it should be possible to define a single set of eigenvectors that could be used to reconstruct all matrices in the series with high fidelity.
3. Finally, the eigenvectors should be related with the properties that have already been identified as important for measuring similarity between amino acids, such as hydrophobicity, molecular size or electric charge – the current approach is not proposed to invalidate earlier work, but to explain earlier results in a more formal and ordered framework.

To check whether these conditions are indeed fulfilled one needs to define a basic set of eigenvectors that can be used as a reference for comparisons and as a base for reconstruction of all matrices and which can be compared with measured properties of amino acids. To this end the eigenvectors derived from the **B** ^100^ matrix were selected. **B** ^100^ is a high-entropy limit of the BLOSUM series, and hence by choosing it as a reference one can test conservation across wide ranges of information entropies. The same range could possibly be tested by selecting **B** ^30^ matrix, which is the low-entropy limit in the BLOSUM family, however, the **B** ^100^ matrix is obtained using all information contained in BLOCKS database, while very little information is left in **B** ^30^ matrix. Following the notation for matrices, the set of eigenvectors derived from the **B** ^x^ (or more precisely from the **P** ^x^) is further denoted as **E** ^x^, for example the **E** ^62^ refers to the set of eigenvectors obtained from **B** ^62^ matrix.

### Conservation of eigenvectors

To examine the conservation of the eigenvectors across the entire range of BLOSUM matrices all matrices were decomposed into sums of the G-matrix and P-matrix. Then for each **B** ^x^ matrix the **E** ^x^ was derived from **P** ^x^. Then the correlations between all eigenvectors in **E** ^x^ with all eigenvectors in **E** ^100^ were computed. Finally the correspondence between vectors in the test set and eigenvectors from **B** ^100^ was established, that is, for each vector 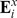 the vector 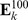 was found that had the highest correlation value. One should note that it is not required that corresponding eigenvectors are identically ordered in **E** ^x^ and **E** ^100^ sets. The order corresponds to the order of eigenvalues that can change between matrices. Hence it may be necessary to compute more than one correlation for each eigenvector in a set, to be certain that no important correlation has been overlooked. The threshold that was used to identify significant correlations was set at 0.5 for the square of correlation.

### Reconstruction of similarity matrices

The reconstruction procedure was very simple. Each matrix **B** ^x^ was reconstructed as a sum of **G** ^x^ and **P** ^x^. The P-matrix is reconstructed using the following formula:
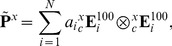
(17)where 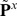 is the reconstructed **P** ^x^, *a_i_* is a parameter, and the 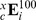 is the eigenvector of the **P** ^100^ matrix that corresponds to i-th eigenvector of the **P** ^x^ matrix, *N* varies between 0 and 8. The initial value of *a_i_* is set equal to the i-th eigenvalue of **P** ^x^ matrix – 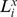 and the minimization of the difference between **P** ^x^ and 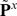 is performed using *a_i_*s as free parameters with the help of the Newton method.

### Model selection

It is a well-known phenomenon that by increasing the number of parameters in the model, one increases the fidelity of the model for known data but also increases the chance of over-fitting. Thus, at some point, by adding extra parameters to the model, fidelity actually decreases. This is a well-known problem when a model is developed on a relatively small sample and is then applied to predict results for unseen data. The rule of thumb is that the number of data points used for model building has to be at least one order of magnitude larger than the number of parameters. A more formal approach was proposed in the form of the so-called Akaike Information Criterion (AIC) [27] and Bayesian Information Criterion (BIC) [28]. The BIC criterion is usually more restrictive than the AIC and therefore it was used in the current study. The formula for BIC is:
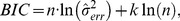
(18)where n is the number of degrees of freedom, k is the number of parameters in the model, and 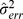 is the estimate of error variance of the model. The first term usually decreases with increased number of parameters, whereas the second increases. The first is the optimistic estimate of the model quality, whereas the second represents pessimistic correction that takes into account the increased ability of over fitting with increasing number of parameters. The balance between these terms usually gives a function with a minimum. To select the best model, one computes the BIC function for a series of models and then selects the one with the minimal BIC. This procedure was used to establish the number of eigenvectors used for reconstruction of the **P** ^x^ matrices, the number of degrees of freedom was the number of the off-diagonal elements in the similarity matrices (190).

### Correspondence between properties and eigenvectors

The amino acid properties measured experimentally and derived by theoretical means are collected in the aaindex database [17]. This database contains descriptors of different origins – some properties are measured experimentally (such as free energy of hydration), some other are elementary properties of amino acids (such as number of bonds in the side-chain), yet some other are basic properties estimated with theoretical methods (accessible surface area), whereas some are derived from statistical data collected from known protein structures. We do not consider the last category as fundamental and elementary and hence these properties were manually filtered out from the database before analysis, leaving the reduced set consisting of 149 properties. These properties were normalized and the correlation coefficients with the eigenvectors were computed.

The mean values of the eigenvectors of BLOSUM matrices are very close to zero. There is one exception to this observation – the first eigenvector is has an average value 0.223 that is almost equal to the square root of 1/20. Moreover, the deviations from the mean value are small – the standard deviation is 0.022. Therefore, it follows from Eq. 17 that this vector contributes mostly to the average value of the similarity matrix, with small contribution to the variance of the model. Taking aside the first eigenvector, for the remaining eigenvectors 2–20 the correlation of an eigenvector with a property is equivalent with their scalar product. Therefore, the set of 19 correlations of a given property with eigenvectors 2–19 is a set of direction coefficients in 19-dimensional space spanned on these eigenvectors.

Interpretation in the opposite direction, i.e. interpreting eigenvectors in terms of known amino acid properties is less obvious, because properties do not constitute the orthonormal set. Nevertheless, the large value of the square of the scalar product between a property and an eigenvector indicates that both vectors are strongly related. Another useful measure is the sum of squares of scalar products of a given property with a range of eigenvectors – a high value (approaching one) indicates that the given property can be entirely decomposed into contributions from this set and no other eigenvectors contribute significantly.

## Results

The entire procedure described above was performed for decomposition of the BLOSUM matrices using both Eq. 8 and Eq. 11. Results were similar, however, models obtained with the help of the Eq. 11 were consistently and significantly better that those obtained from the Eq. 8. Therefore, only results for the former are presented here.

### Conservation of eigenvectors

**Figure 1:**
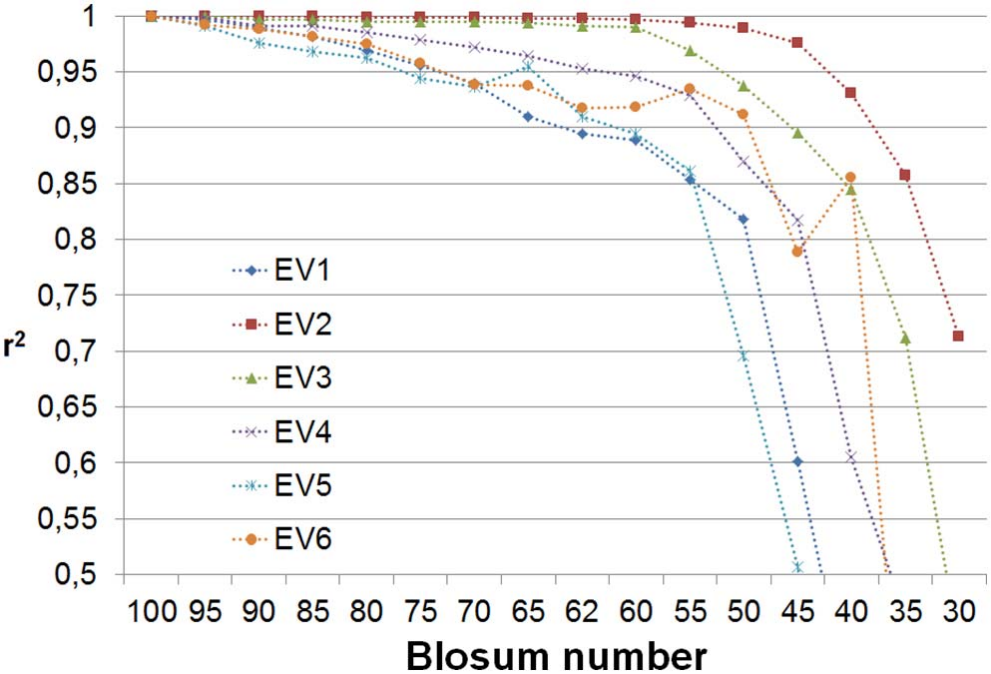
Squares of correlation coefficients between eigenvectors obtained from B^x^ matrix with the eigenvectors obtained from the B^100^ matrix.

The first test of the approach described in the previous section is the conservation of eigenvectors – the ones obtained for the entire range of BLOSUM matrices should be similar. Figure 1 displays the squares of correlations between eigenvectors 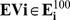 and 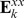, where 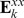 is the eigenvector with highest correlation with EVi, i = 1…6. One can see that, contrary to initial expectations, the best-conserved eigenvector is not the one that corresponds to the largest eigenvalue, namely the EV1. The curve corresponding to EV1 is very close to that of EV5. Nevertheless, the next few eigenvectors follow the expected pattern – the conservation level of eigenvectors is highest for EV2, then for EV3 and EV4. For all eigenvectors the correlation level at first decreases very slowly and then falls rapidly after r^2^ drops below 0.9. The only eigenvector that has relatively high correlation for the entire range of entropies is EV2. It is interesting that correlations are quite good until vectors obtained from **B** ^50^ or **B** ^45^ matrices, which are those with lowest entropy recommended for similarity searches with BLAST. We don’t know whether this is just a coincidence or a consequence of too small information content in these matrices, to make them suitable both for similarity searches and present type of analysis.

### Matrix reconstruction

**Figure 2:**
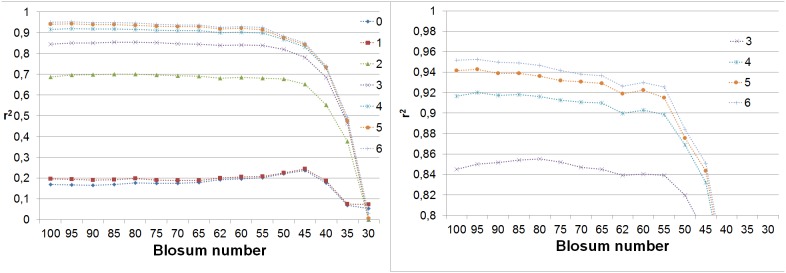
Squares of correlation coefficients between the of-diagonal elements of B^x^ matrices and their reconstructions. The i-th model of each matrix is reconstructed using i eigenvectors. The reconstruction with i = 0 is limited to G-matrix, the remaining models use eigenvectors EVi (i = 1…6). The left panel displays entire range of r^2^, the right panel is a close-up for r^2^ larger than 0.8.

High stability of the eigenvectors for nearly the whole range of entropies is one indicator that indeed they may be good descriptors of properties conserved in evolution. The values of the first six eigenvectors of the BLOSUM100 matrix, which are used further as the base set, are listed in Table 1. The next test undertaken to substantiate the hypothesis was to reconstruct all the similarity matrices from the BLOSUM series using the single basis set derived from the **B** ^100^. The synthetic effects of the reconstruction for the entire series are presented in Figure 2 and Table 2. The first observation is that the model that is based on G-matrix alone explains between 16% and 25% of variability in the BLOSUM series of matrices. This is by definition achieved without taking into account any pair-specific information. The lower values correspond to high entropy matrices and then the contribution of the non-specific part increases with decreasing entropy of the matrix until it reaches maximum for **B** ^45^. Then it rapidly falls down. The next interesting observation is that the first eigenvector contributes very little to the variability of the matrix. The explanation is straightforward – the first eigenvector has very similar values for all amino acids, as can be seen in Table 1. The first term in the sum in Eq. 17 corresponds to adding the average value to all elements of the reconstructed similarity matrix. The by far highest contribution to the matrix variability is due to the second eigenvector. The model built with G-matrix and the two first eigenvectors explains nearly 70% of the variability in most matrices. This value falls below 60% only for the three matrices with lowest entropy (**B** ^40^, **B** ^35^, **B** ^30^) and for **B** ^30^ it falls down to zero. After adding the third eigenvector the model explains more than 80% of the variability in most of the matrices. The fourth and fifth eigenvectors increase the model fidelity above 90%, whereas the sixth eigenvector adds a relatively small contribution explaining roughly 1% of variability in BLOSUM matrices.

**Table 1:** Eigenvectors of BLOSUM100 matrix.

Amino Acid	EV1	EV2	EV3	EV4	EV5	EV6
A	0.2167	0.0494	0.2345	0.2496	0.3504	0.0594
R	0.2217	0.1822	−0.1103	−0.2950	0.3594	−0.0954
N	0.2220	0.2619	−0.0224	0.1362	−0.3214	−0.0092
D	0.2335	0.3024	0.0104	0.0710	−0.5125	0.0622
C	0.1740	−0.1616	0.1909	0.3443	0.0324	−0.0252
Q	0.2156	0.1984	−0.1189	−0.2772	0.0783	−0.0669
E	0.2347	0.2812	−0.0080	−0.1963	−0.2618	−0.0964
G	0.2129	0.1650	0.0657	0.3523	0.1910	−0.1504
H	0.1973	0.1346	−0.3638	−0.0206	−0.0967	0.3454
I	0.2767	−0.3426	0.2297	−0.1713	−0.2171	0.0951
L	0.2155	−0.3112	0.1009	−0.2111	−0.0415	−0.0337
K	0.2406	0.2288	−0.0198	−0.3078	0.2746	0.1417
M	0.2452	−0.2356	0.0993	−0.2999	0.0457	−0.1522
F	0.2198	−0.3142	−0.3134	0.1276	−0.0529	0.3275
P	0.2284	0.1107	0.1136	0.0077	0.2651	−0.0406
S	0.2188	0.1508	0.1584	0.3135	0.0865	0.0028
T	0.2099	0.0324	0.2244	0.1616	−0.0884	0.0402
W	0.2059	−0.2112	−0.3941	0.1733	0.1718	0.3719
Y	0.2121	−0.1937	−0.4991	0.1724	−0.0492	−0.7234
V	0.2515	−0.2746	0.2843	−0.0978	−0.1181	−0.0385

**Table 2:** The variance explained by the eigenvectors in reconstructed BLOSUM matrices.

	Number of eigenvectors					
Blosum	0	1	2	3	4	5
100	16,9%	19,7%	68,8%	84,5%	91,7%	94,2%
95	16,8%	19,5%	69,7%	85,0%	92,0%	94,3%
90	16,7%	19,2%	69,8%	85,2%	91,7%	93,9%
85	17,0%	19,4%	70,1%	85,4%	91,8%	93,9%
80	17,8%	19,9%	70,1%	85,5%	91,6%	93,6%
75	17,5%	19,1%	69,6%	85,2%	91,3%	93,2%
70	17,5%	19,0%	69,4%	84,7%	91,1%	93,0%
65	17,9%	19,0%	69,1%	84,5%	91,0%	92,9%
62	19,3%	20,1%	68,2%	83,9%	90,0%	91,9%
60	19,7%	20,5%	68,5%	84,0%	90,3%	92,2%
55	20,2%	20,7%	68,1%	83,9%	89,8%	91,5%
50	22,1%	22,6%	67,7%	82,0%	86,9%	87,6%
45	23,8%	24,4%	65,3%	78,2%	83,2%	84,3%
40	17,7%	18,7%	55,2%	68,5%	72,9%	73,3%
35	6,9%	7,6%	37,8%	46,3%	47,3%	47,6%
30	5,4%	7,4%	-	-	-	-

The fraction of variance in an original BLOSUM matrix explained by reconstruction carried out with the eigenvectors of BLOSUM100 matrix. The number of eigenvectors varied between 0 (the reconstruction using the non-specific G-matrix only) and 5.

**Figure 3:**
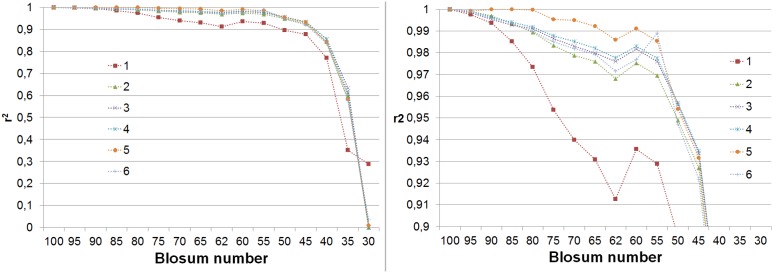
The relative quality of the reconstructed matrices. The value on Y-axis is a fraction of best possible reconstruction achieved by the model. It is estimated as a fraction of r^2^ achieved in reconstruction with eigenvectors derived from **B** ^100^ and r^2^ obtained in reconstruction with its own eigenvectors. The left panel displays entire range of Y, the right panel is a close-up for Y larger than 0.9.

The proposed model of BLOSUM matrices works well for matrices in the range spanned by **B** ^100^ and **B** ^45^, its quality decreases for **B** ^40^ and **B** ^35^ matrices and it fails for **B** ^30^ matrix. It is clearly visible on Figure 3, which displays the quality of the models of BLOSUM matrices reconstructed with eigenvectors of **B** ^100^ matrix relative to the reconstruction of the same matrices with their own eigenvectors. The reconstructions are close to the best possible for matrices between **B** ^100^ and **B** ^55^, the quality decreases considerably for matrices **B** ^50^ and **B** ^45^ and then falls rapidly for remaining matrices.

**Figure 4:**
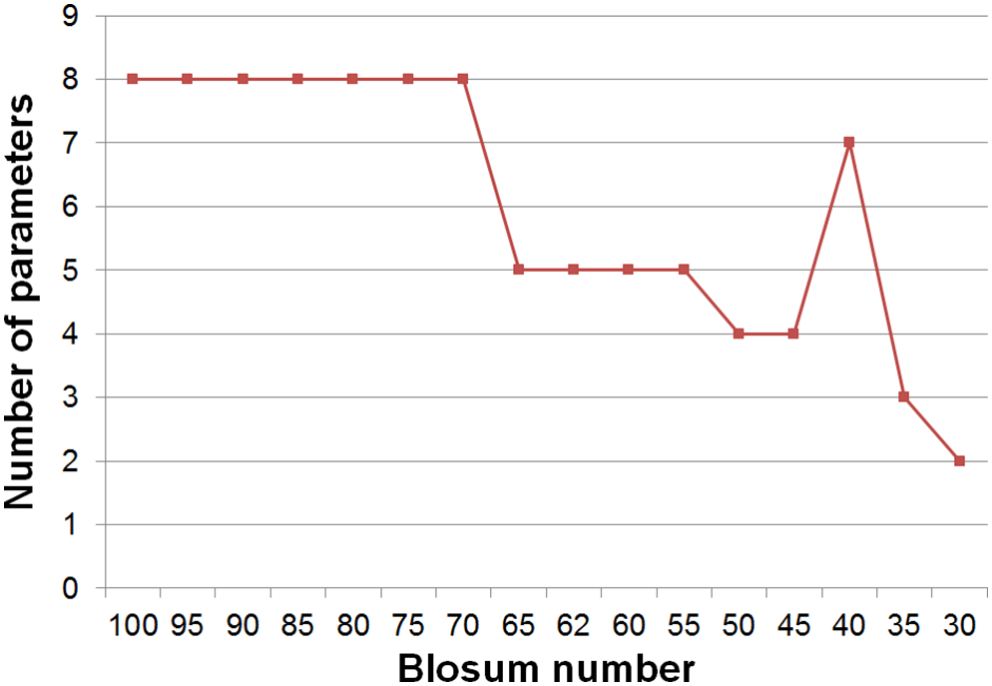
The optimal number of eigenvectors for reconstruction of the BLOSUM matrices. The optimal number of eigenvectors was obtained with the help of the Bayesian Information Criterion.

The simple use of BIC for selection of the models for matrices leads to results that are certainly too optimistic for high entropy matrices, down to BLOSUM 70, see Figure 4. The BIC index was computed for models built with up to 8 eigenvectors and for all these matrices the maximum was achieved at 8 eigenvectors. It is more likely that this result reflects high similarity between high-entropy matrices than conservation of important properties. For medium entropy matrices (**B** ^65^, **B** ^62^, **B** ^60^, **B** ^55^) the BIC index falls to 5, then it falls to 4 for **B** ^50^ and **B** ^45^ and to 3 for **B** ^35^. The spike at **B** ^40^ is most probably a numerical artefact. Finally the value for **B** ^30^ is meaningless, since the quality of model is very low. The values of the optimised coefficients in the Eq. 17 that were used to reconstruct BLOSUM matrices are presented in Table 3.

**Table 3:** Optimised coefficients for matrix reconstruction for final models.

BLOSUM matrix	N	E1	E2	E3	E4	E5
100	5	−23.28	13.61	7.78	5.07	2.98
95	5	−22.17	13.44	7.49	4.89	2.78
90	5	−21.55	13.05	7.26	4.57	2.58
85	5	−20.06	12.67	7.05	4.37	2.46
80	5	−17.55	12.01	6.75	4.07	2.30
75	5	−14.10	11.50	6.46	3.87	2.16
70	5	−12.90	10.96	6.14	3.77	2.09
65	5	−9.49	10.31	5.79	3.59	1.95
62	5	−7.64	9.41	5.45	3.24	1.80
60	5	−12.54	10.05	5.37	3.36	−2.20
55	5	−4.69	8.19	4.82	2.80	1.48
50	4	−3.46	7.26	3.96	2.27	-
45	4	−3.77	6.02	3.25	2.02	-
40	4	−5.59	5.40	3.29	1.79	-
35	3	−7.06	6.25	3.04	-	-
30	2*	−5.62	4.21	-	-	-

The N refers to the number of eigenvectors used in the final reconstruction, whereas Ei are the optimised coefficients. In the case of BLOSUM30 the eigenvalues of BLOSUM30 are used.

**Figure 5:**
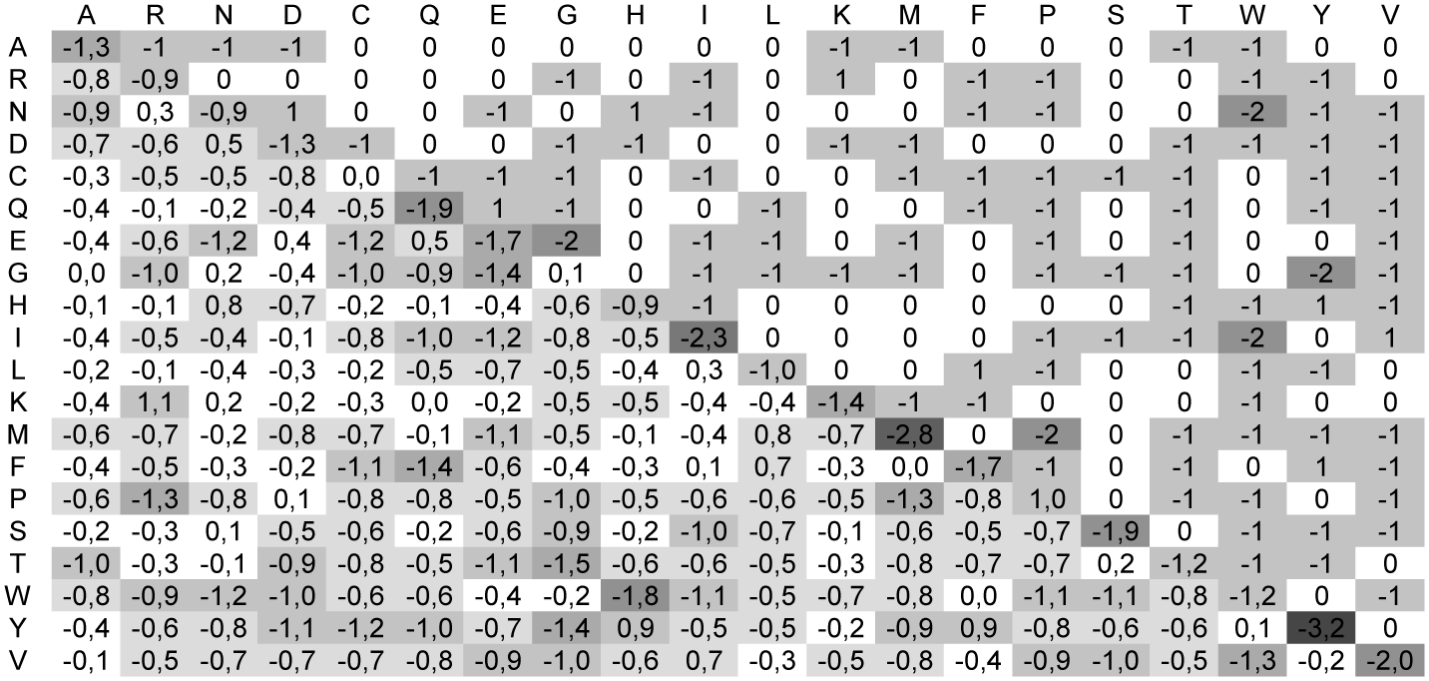
The differences between original BLOSUM62 matrix and its reconstruction. The matrix was reconstructed using G-matrix and 5 eigenvectors derived from BLOSUM100 matrix. The triangle above diagonal displays differences between matrices scaled to half bit units and rounded, the diagonal and triangle below displays differences between matrices scaled to half bit units and not rounded. The shades of gray correspond to the differences.

An example of the results of the reconstruction procedure is shown in Figure 5. It displays differences between the elements of the BLOSUM62 matrix and their reconstructions. The upper off-diagonal part holds values obtained from differences between integer representation of the matrix, whereas the lower right part holds the differences between non-rounded variants of the matrices. The standard representation of this matrix is in half bit units and rounded to integer. 76 percent of the off-diagonal elements are reproduced within 0.25 bit from the original value 93 percent within 0.5 bit and 99 percent within 0.75 bit. The figures that illustrate full sequence of reconstructions for **B** ^62^ as well as for **B** ^80^ and **B** ^45^ matrices can be found in Figures S1–S20. Both, numerical value of correlation between original and reconstructed matrices, as well as visual inspection of the differences, show that the method works very well.

The final question is how the new synthetic properties compare with the physical properties known to correlate with AASMs. To answer this question the correlations of the five eigenvectors corresponding to highest eigenvalues with properties collected in the aaindex database were computed. As mentioned earlier, the correlation coefficient of the normalized property computed with eigenvector other than the first one is nearly identical with the scalar product.

**Figure 6:**
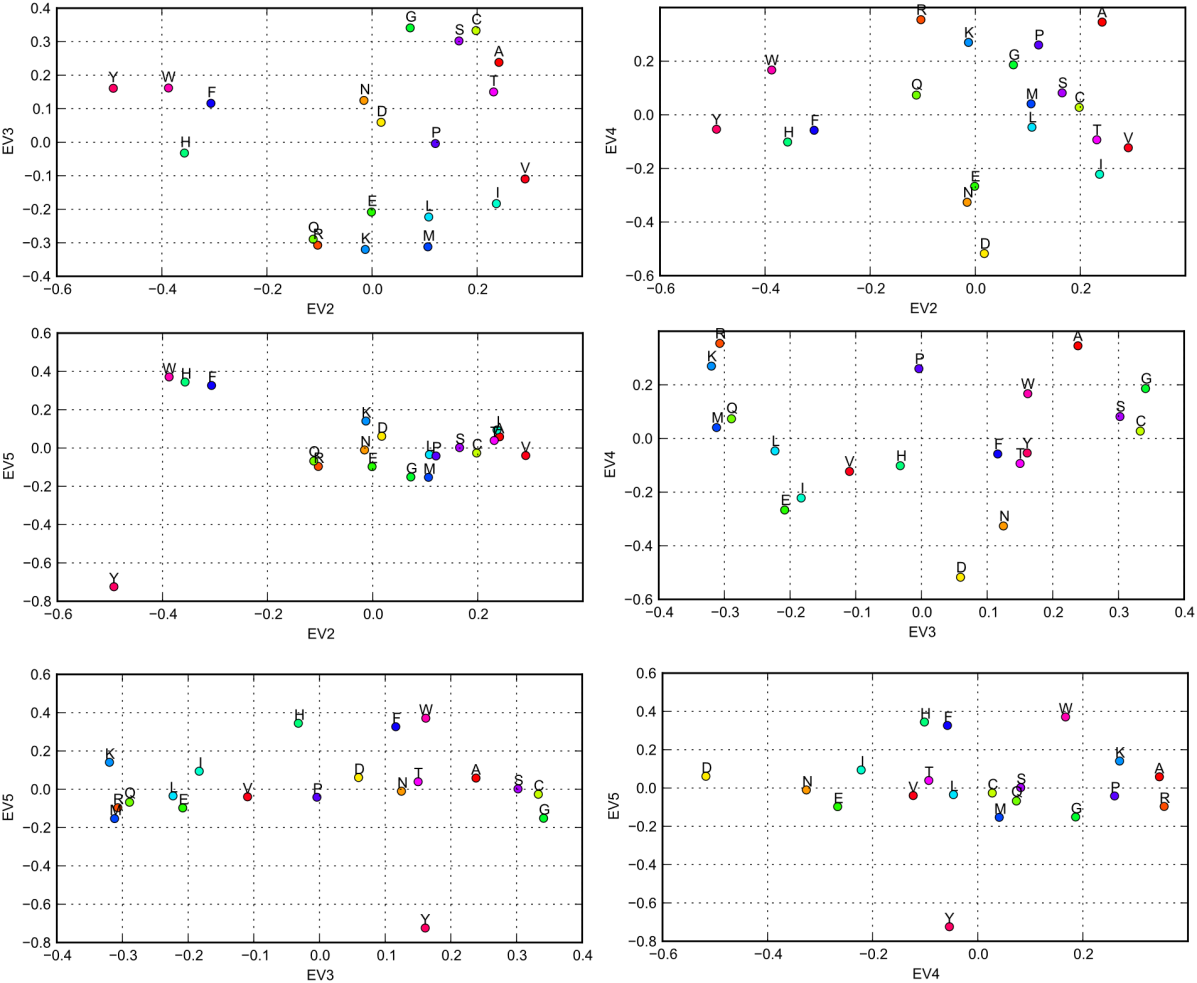
Amino acids in the eigenvector space. Six panels display projections of amino acids on all combinations space spanned on four eigenvectors: EV2, EV3, EV4 and EV5.

The previous results show that meaningful models can be built using the five eigenvectors with largest eigenvalues. The first eigenvector does not contribute significantly to the variability of the matrix, but instead is responsible for elevating its average value. Nevertheless, the correlation coefficients 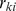 of amino acid **R** *_k_* with eigenvectors EVi were computed for *i* = 1…6. The sixth eigenvector was included to examine whether any properties that are known to be important could be omitted by limiting the eigenvector number to five. Then for each property the fraction of length of the property contained in the 4-dimensional subspace spanned on the set of four eigenvectors (EV2, EV3, EV4, EV5) was computed:
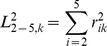
(19)where 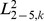 is the sum of squares of correlation coefficients. Two properties with highest correlations with each eigenvector are displayed in the Table 4. The inspection of Table 4 reinforces earlier results. The eigenvectors 2–5 are indeed strongly correlated with properties, which are already known to be connected with AASMs. In particular the EV2 is highly correlated with properties describing hydrophobicity of the residues, EV3 and EV4 are both correlated with various measures of the size of the residues, and EV5 is correlated with residue charge. One may observe that apparently similarity of both size and shape impacts the accepted mutations – this is implied due to the correlation of EV3 and EV4 with different descriptors connected with size. The quite strong correlation of the fifth eigenvector with charge of the residue shows the role of the electrostatics for mutations. Location of amino acids in the four-dimensional space spanned on eigenvectors EV2, EV3, EV4 and EV5 is presented in Figure 6.

**Table 4:** The amino acid properties that highly correlate with the eigenvectors of the BLOSUM100 matrix.

L^2^_2-5_	r_1_^2^	r_2_^2^	r_3_^2^	r_4_^2^	r_5_^2^	r_6_^2^	Property with amino acid index entry name
**0.98**	-	0.92	-	0.02	0.04	-	**Polarity (**GRAR740102)
0.96	0.01	**0.94**	-	-	-	-	**Relative partition energies derived by the Bethe approximation (**MIYS990101**)**
0.81	-	0.01	**0.62**	0.17	-	-	**The number of bonds in the longest chain (**CHAM830106**)**
0.80	-	0.05	**0.62**	0.13	-	-	Molecular weight (FASG760101)
0.73	**0.32**	0.42	0.05	0.21	0.03	0.01	**Partial specific volume (**COHE430101**)**
0.66	0.01	-	0.19	**0.43**	0.01	0.03	**STERIMOL length of the side chain (FAUJ880104)**
0.63	0.01	0.24	0.26	0.02	0.01	0.11	Partition coefficient (Garel et al., 1973)
0.62	-	-	0.02	0.13	0.45	0.01	Isoelectric point (ZIMJ680104)
0.57	-	-	-	0.05	**0.50**	-	**Net charge (**KLEP840101**)**
0.56	-	-	0.12	0.42	0.02	-	Entropy of formation (HUTJ700103)
0.56	0.09	0.13	0.11	0.04	0.15	**0.13**	**Free energy of solution in water, kcal/mole (**CHAM820102**)**
0.46	0.25	0.04	0.29	0.08	0.03	0.02	alpha-CH chemical shifts

The column heads: L^2^_2–5_– The square of length of property vector projected onto hyperplane spanned on the eigenvectors 2–5; r_i_^2^ (i = 1–6) – the square of correlation coefficient with i-th eigenvector. The values highlighted in boldface are a maximum in the column; the corresponding property is also highlighted in boldface. The values above 0.10 are underlined.

Two remaining eigenvectors from those examined, namely EV1 and EV6 are not correlated with selected properties to a substantial extent. For both these eigenvectors the properties that correlate most strongly with them correlate more strongly with other eigenvectors. What is more, the maximal correlation is rather small – in particular in the case of the sixth eigenvector. This finding independently confirms that five eigenvectors are a good choice for building the amino acid matrices. While the first eigenvector does not contribute much to the variance it does contribute significantly to the average value of reconstructed matrices and hence should be included. However, the sixth eigenvector neither contributes much to variance nor is it correlated with any known property to a significant extent.

It is interesting to notice that the property that is nearly perfectly aligned with the hyperplane spanned on eigenvectors EV2, EV3, EV4 and EV5 is polarity, originally introduced by Grantham in 1974 [29] to build a distance matrix for amino acids.

For 122 of amino acid properties, out of 149 that were examined within the current study, most of their variability is confined to the hyperplane spanned on eigenvectors EV2…EV5. The sum of the squares of correlation coefficients 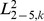 of these properties with eigenvectors EV2…EV5 is larger than 0.5. Within this set 83 properties have 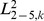 higher than 0.7 and 54 have it higher than 0.8. This result clearly shows that properties that were researched in the literature are strongly biased towards the hyperspace spanned on the eigenvectors EV2…EV5.

## Discussion

The idea of using eigenvalue decomposition for analysis of the similarity matrices is not new. Kinjo and Nishikawa [30] examined a series of matrices derived from mutation data in a manner similar to BLOSUM searching for amino acid properties that are conserved in evolution. They discovered a transition between two modes – for closely related proteins the mutability was considered most important, whereas for distant proteins the hydrophobicity became more important. The transition was observed for sequence identity around 30–35 percent, which is the same region where quality of the matrix reconstruction with the model proposed in the current study rapidly deteriorates and ultimately fails for BLOSUM30 matrix.

The singular value decomposition of matrices, which for square matrices is equivalent to eigenvalue decomposition, was used recently by Zimmerman and Gibrat [31] in their analysis of amino acid properties conserved in evolution. They applied it as an intermediate step in their method aiming at representing amino acids matrices as scalar products of amino acid property vectors. In their approach they first obtained a set of vectors and then correlated them with selected amino acid properties. They used 17 properties and estimated their contributions to BLOSUM and PAM similarity matrices. A similar approach was proposed previously by Pokarowski et al. [32] who aimed at reconstruction of similarity matrices from scalar products of arbitrary vectors.

Our study differs from previous efforts in two key aspects. Firstly, only mutability and four mutually orthogonal fundamental properties are used in the model for all matrices, hence the model developed in the current study is more parsimonious than earlier approaches. These properties are derived as eigenvectors of pairwise-specific part of the BLOSUM100 matrix. Moreover, our approach allows for a more precise estimate of contributions to the substitution matrices of various properties conserved in the evolution. In our analysis we explicitly separate the effects of pair-wise similarities between amino acids from those arising from mutability, which is a property of a single amino acid. One should stress, however, that mutability is a special property. As can be seen in Eq. 3 it depends explicitly on similarities to all other amino acids, hence it is a derivative of fundamental properties. Indeed, 70 percent of variance in mutability is explained by the five first eigenvectors. Nevertheless, including mutability explicitly in the model allows taking into account the collective effects of the remaining eigenvectors.

The selection of BLOSUM100 matrix as a base was arbitrary, but the results don’t depend on the selection of a particular matrix. An identical analysis was performed using BLOSUM62 as a base, with nearly identical results. The results were actually slightly better for the low entropy matrices in that case, nevertheless, we decided to present the results in the current form because selection of BLOSUM100 is a more conservative approach – the distances from low entropy matrices to BLOSUM100 are higher than those to BLOSUM62 matrix.

The newly derived synthetic properties are both orthogonal to each other and are relevant for interactions between amino acids. Hence they are better suited to be used in analyses of protein sequences than either standard physical and chemical properties or vectors arising from principal component analysis. The physical and chemical properties are neither orthogonal, nor are they directly connected with amino acid mutations. While principal components of the property space are orthogonal, their interpretation is difficult and they strongly depend on the composition of the database. The eigenvectors of the BLOSUM100 matrix are derived from the data on amino acid mutations, are orthogonal and interpretable in terms of known properties. The proposed model allows to attribute up to 94% of the variability of the off-diagonal part of similarity matrices to well defined factors.

It is unlikely that more sophisticated models could improve our results significantly. The success of sequence analysis methods that are based on the position specific similarity matrices [15], as well as the demonstrated dependence of mutation probability on local context [33], [34] shows the limits of general models. The methodology developed in the current study can be also used for analysis of AASMs derived for more specialised applications, such as, for example, analysis of membrane proteins [35] or properties in local protein environments [33] and possibly also for improving current sequence homology search tools.

## Supporting Information

Figure S1 Reconstruction of the BLOSUM80 matrix – stage 0, G-matrix and no eigenvectors. The 6 stages of reconstruction of BLOSUM80 matrix with eigenvectors from BLOSUM100 are presented in Figures S1-S6. The stage 0 involves the non-specific G-matrix only, stage 1 involves the first eigenvector of BLOSUM100, and k-th stage involves k eigenvectors. For each matrix the triangle above diagonal displays differences between matrices scaled to half bit units and rounded, the diagonal and triangle below displays differences between matrices scaled to half bit units and not rounded. The shades of gray correspond to the differences. The gray scale is presented in Figure S7.
(TIF)

Figure S2 Reconstruction of the BLOSUM80 matrix – stage 1, G-matrix and one eigenvector.
(TIF)

Figure S3 Reconstruction of the BLOSUM80 matrix – stage 2, G-matrix and two eigenvectors.
(TIF)

Figure S4 Reconstruction of the BLOSUM80 matrix – stage 3, G-matrix and three eigenvectors.
(TIF)

Figure S5 Reconstruction of the BLOSUM80 matrix – stage 4, G-matrix and four eigenvectors.
(TIF)

Figure S6 Reconstruction of the BLOSUM80 matrix – stage 5, G-matrix and five eigenvectors.
(TIF)

Figure S7 The scale for Figures S1-S6. The shades of gray correspond to the differences between original and reconstructed matrices. The maximal value for the scale is obtained as the absolute value of the off-diagonal elements of the original BLOSUM80 matrix, the minimal value is zero. The scale is divided equally into ten intervals.
(TIF)

Figure S8 Reconstruction of the BLOSUM62 matrix – stage 0, G-matrix and no eigenvectors. The 6 stages of reconstruction of BLOSUM62 matrix with eigenvectors from BLOSUM100 are presented in Figures S8–S13. The stage 0 involves the non-specific G-matrix only, stage 1 involves the first eigenvector of BLOSUM100, and k-th stage involves k eigenvectors. For each matrix the triangle above diagonal displays differences between matrices scaled to half bit units and rounded, the diagonal and triangle below displays differences between matrices scaled to half bit units and not rounded. The shades of gray correspond to the differences. The gray scale is presented in Figure S14.
(TIF)

Figure S9 Reconstruction of the BLOSUM62 matrix – stage 1, G-matrix and one eigenvector.
(TIF)

Figure S10 Reconstruction of the BLOSUM62 matrix – stage 2, G-matrix and two eigenvectors.
(TIF)

Figure S11 Reconstruction of the BLOSUM62 matrix – stage 3, G-matrix and three eigenvectors.
(TIF)

Figure S12 Reconstruction of the BLOSUM62 matrix – stage 4, G-matrix and four eigenvectors.
(TIF)

Figure S13 Reconstruction of the BLOSUM62 matrix – stage 5, G-matrix and five eigenvectors.
(TIF)

Figure S14 The scale for Figures S8–S13. The shades of gray correspond to the differences between original and reconstructed matrices. The maximal value for the scale is obtained as the absolute value of the off-diagonal elements of the original BLOSUM62 matrix, the minimal value is zero. The scale is divided equally into ten intervals.
(TIF)

Figure S15 Reconstruction of the BLOSUM45 matrix – stage 0, G-matrix and no eigenvectors. The 5 stages of reconstruction of BLOSUM45 matrix with eigenvectors from BLOSUM100 are presented in Figures S15–S19. The stage 0 involves the non-specific G-matrix only, stage 1 involves the first eigenvector of BLOSUM100, and k-th stage involves k eigenvectors. For each matrix the triangle above diagonal displays differences between matrices scaled to 1/3 bit units and rounded, the diagonal and triangle below displays differences between matrices scaled to 1/3 bit units and not rounded. The shades of gray correspond to the differences. The gray scale is presented in Figure S20.
(TIF)

Figure S16 Reconstruction of the BLOSUM45 matrix – stage 1, G-matrix and one eigenvector.
(TIF)

Figure S17 Reconstruction of the BLOSUM45 matrix – stage 2, G-matrix and two eigenvectors.
(TIF)

Figure S18 Reconstruction of the BLOSUM45 matrix – stage 3, G-matrix and three eigenvectors.
(TIF)

Figure S19 Reconstruction of the BLOSUM45 matrix – stage 4, G-matrix and four eigenvectors.
(TIF)

Figure S20 The scale for Figures S15–S19. The shades of gray correspond to the differences between original and reconstructed matrices. The maximal value for the scale is obtained as the absolute value of the off-diagonal elements of the original BLOSUM45 matrix, the minimal value is zero. The scale is divided equally into ten intervals.
(TIF)
