# Supplementary figures and images for: Amino Acid Properties Conserved in Molecular Evolution
Source: PLoS One. 2014 Jun 26;9(6):e98983. doi: 10.1371/journal.pone.0098983 (PMC4072533; doi:10.1371/journal.pone.0098983)

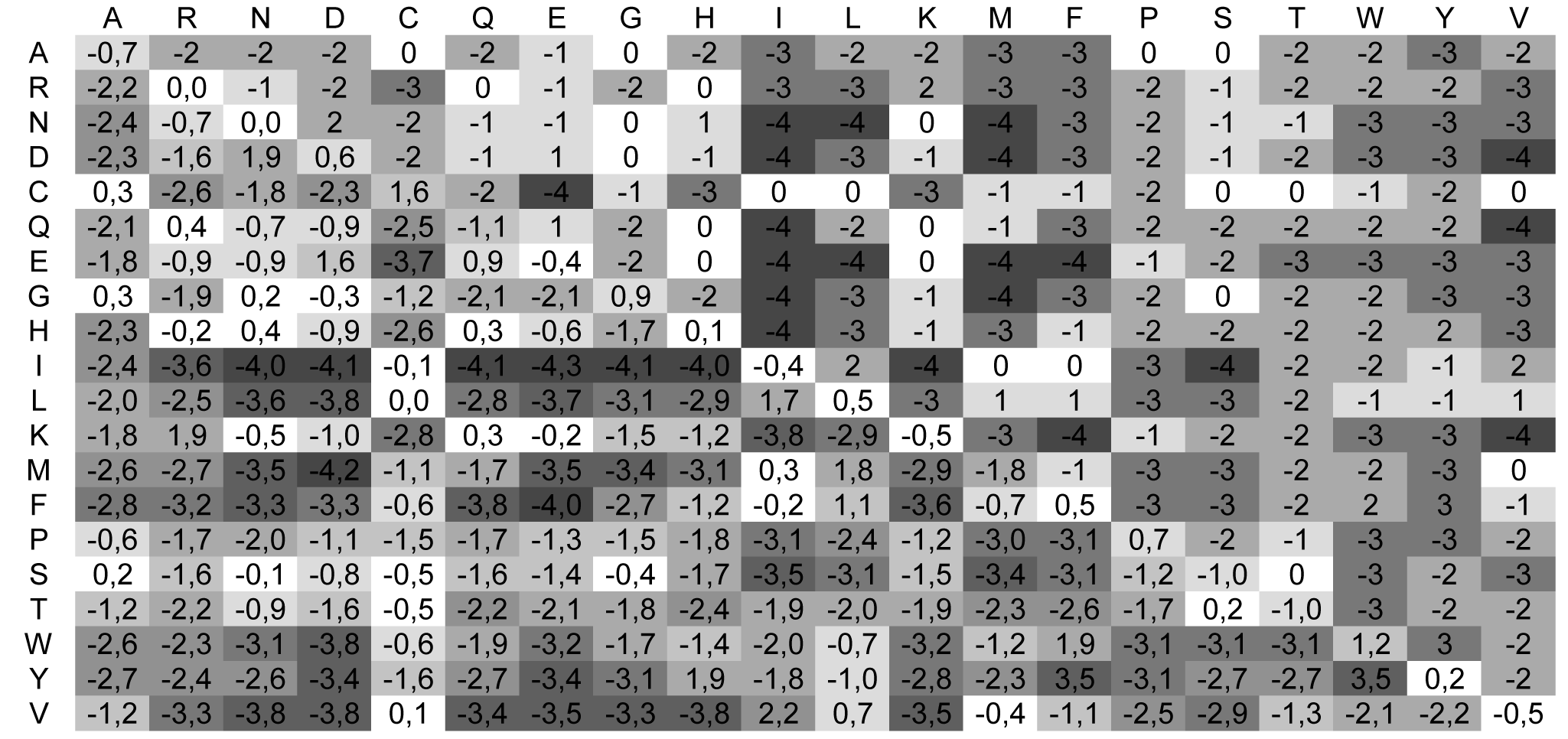

Supplement: Figure S1 — Reconstruction of the BLOSUM80 matrix – stage 0, G-matrix and no eigenvectors. The 6 stages of reconstruction of BLOSUM80 matrix with eigenvectors from BLOSUM100 are presented in Figures S1-S6. The stage 0 involves the non-specific G-matrix only, stage 1 involves the first eigenvector of BLOSUM100, and k-th stage involves k eigenvectors. For each matrix the triangle above diagonal displays differences between matrices scaled to half bit units and rounded, the diagonal and triangle below displays differences between matrices scaled to half bit units and not rounded. The shades of gray correspond to the differences. The gray scale is presented in Figure S7. (TIF) [file pone.0098983.s001.tif]

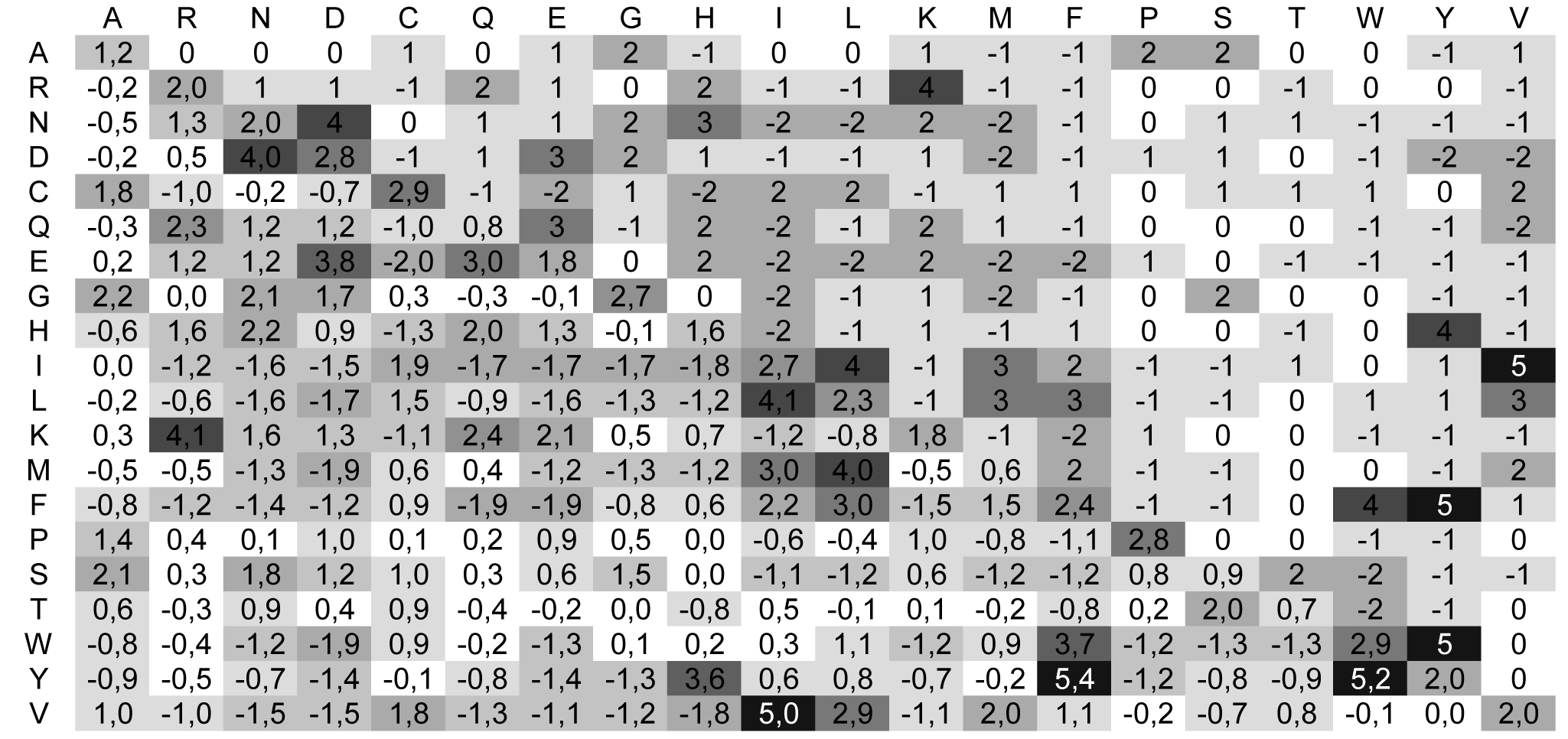

Supplement: Figure S2 — Reconstruction of the BLOSUM80 matrix – stage 1, G-matrix and one eigenvector. (TIF) [file pone.0098983.s002.tif]

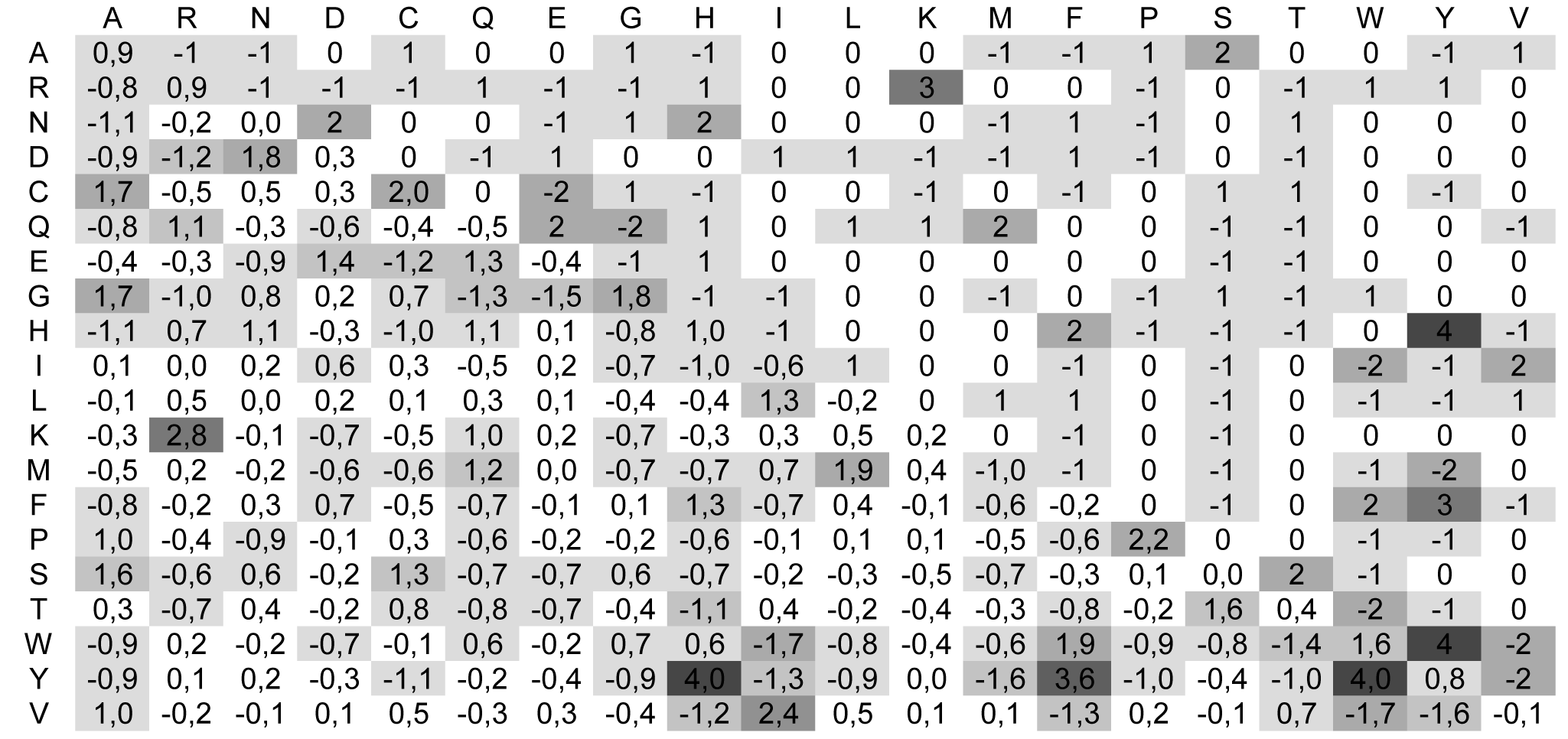

Supplement: Figure S3 — Reconstruction of the BLOSUM80 matrix – stage 2, G-matrix and two eigenvectors. (TIF) [file pone.0098983.s003.tif]

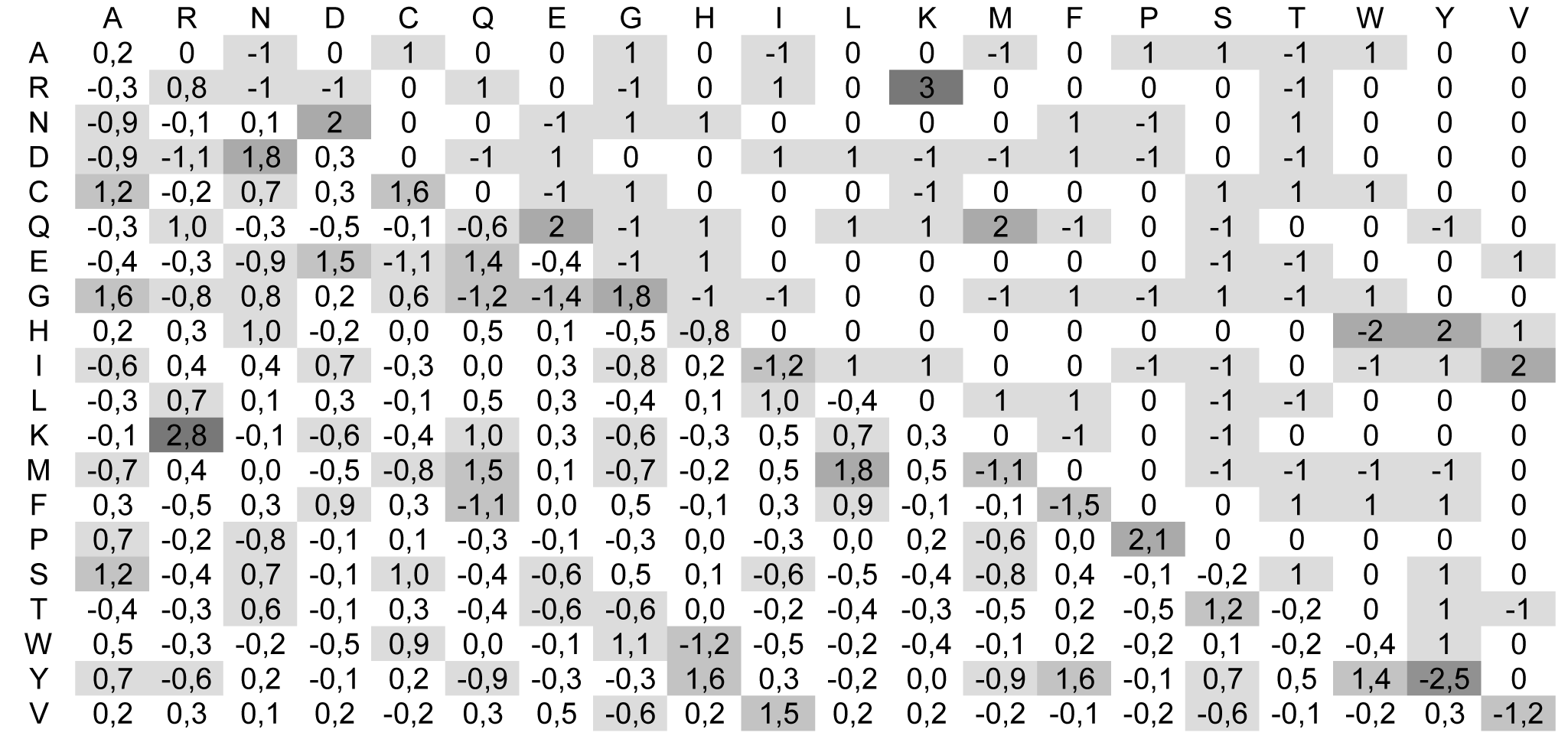

Supplement: Figure S4 — Reconstruction of the BLOSUM80 matrix – stage 3, G-matrix and three eigenvectors. (TIF) [file pone.0098983.s004.tif]

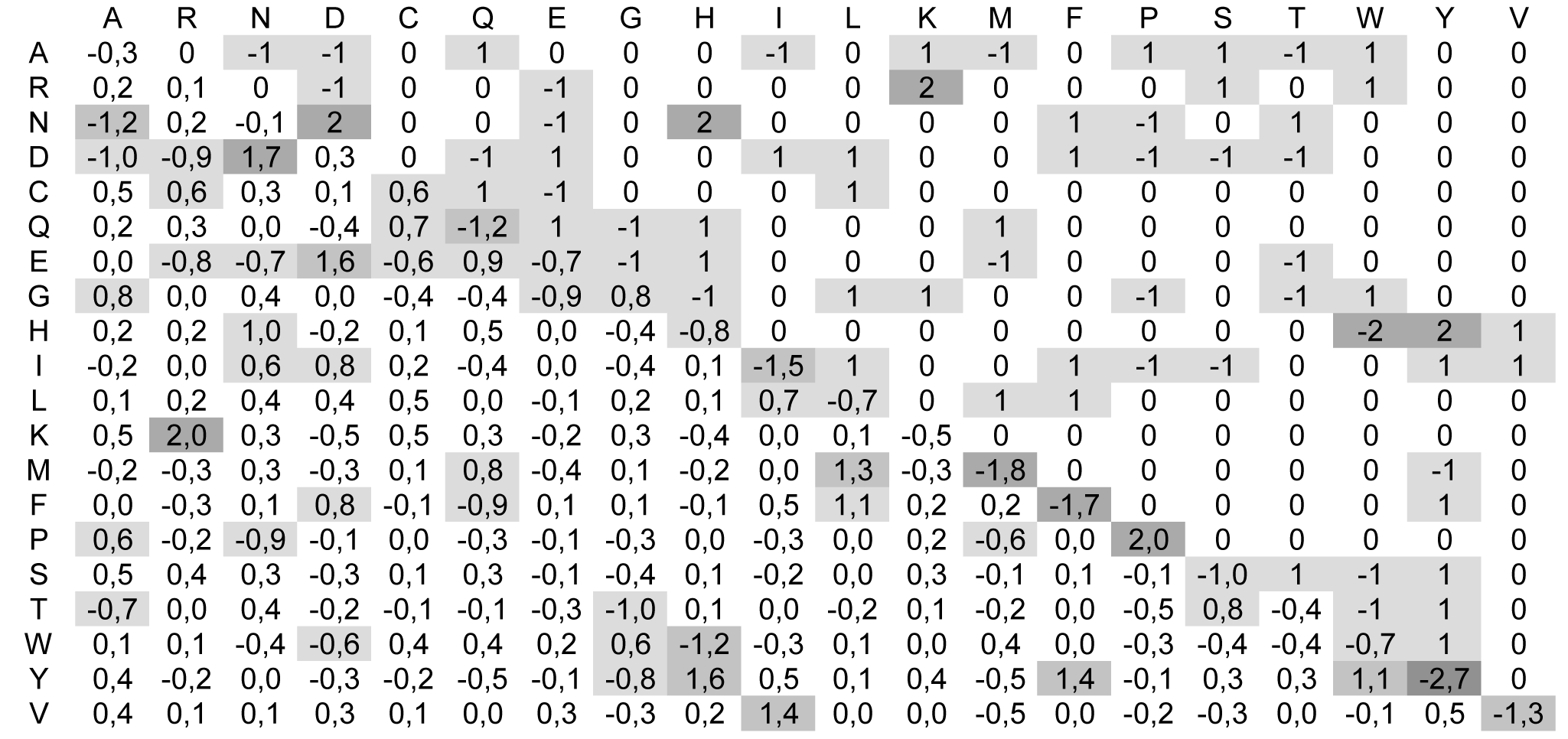

Supplement: Figure S5 — Reconstruction of the BLOSUM80 matrix – stage 4, G-matrix and four eigenvectors. (TIF) [file pone.0098983.s005.tif]

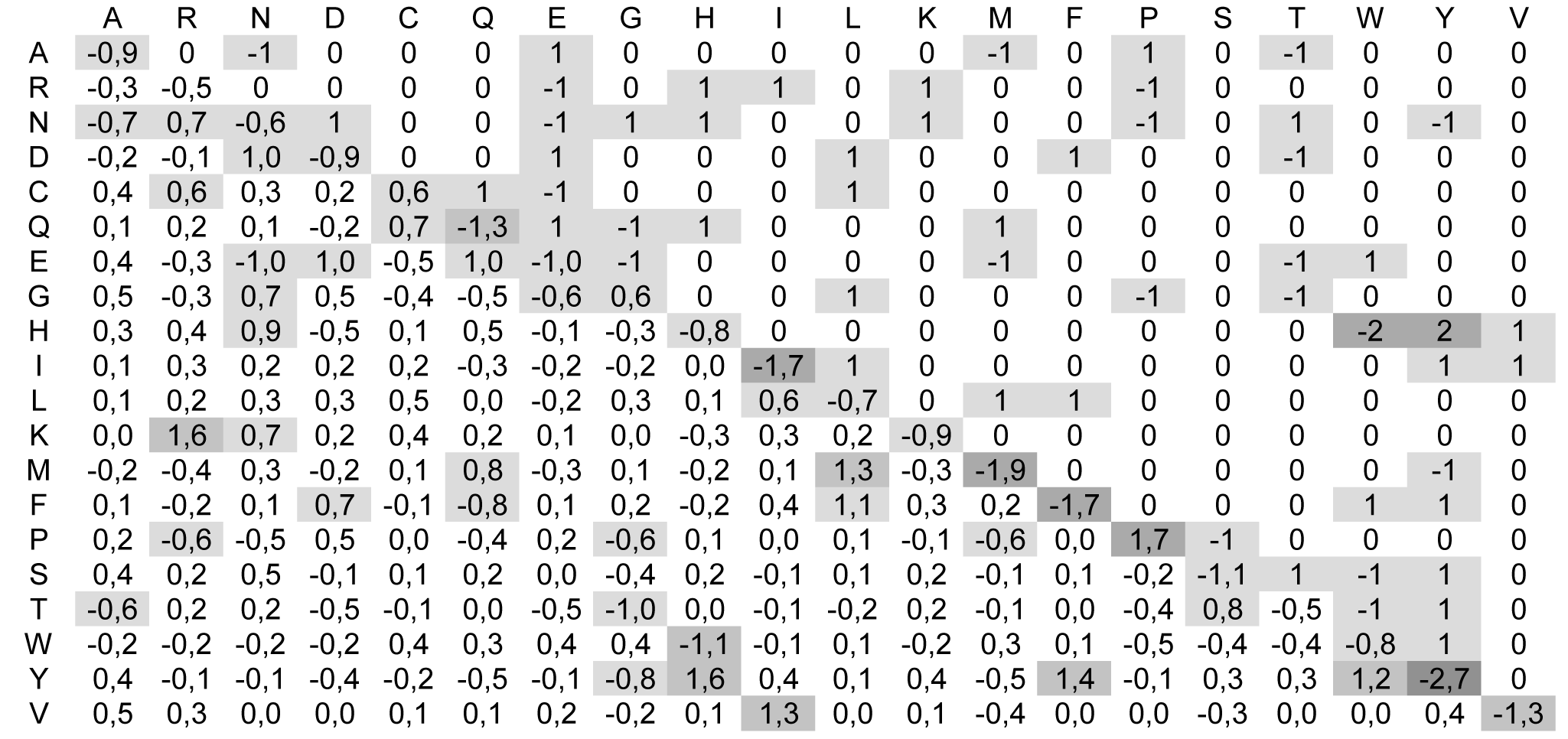

Supplement: Figure S6 — Reconstruction of the BLOSUM80 matrix – stage 5, G-matrix and five eigenvectors. (TIF) [file pone.0098983.s006.tif]

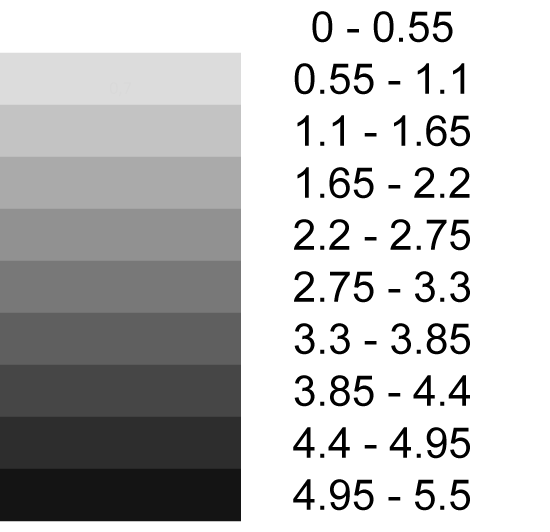

Supplement: Figure S7 — The scale for Figures S1-S6. The shades of gray correspond to the differences between original and reconstructed matrices. The maximal value for the scale is obtained as the absolute value of the off-diagonal elements of the original BLOSUM80 matrix, the minimal value is zero. The scale is divided equally into ten intervals. (TIF) [file pone.0098983.s007.tif]

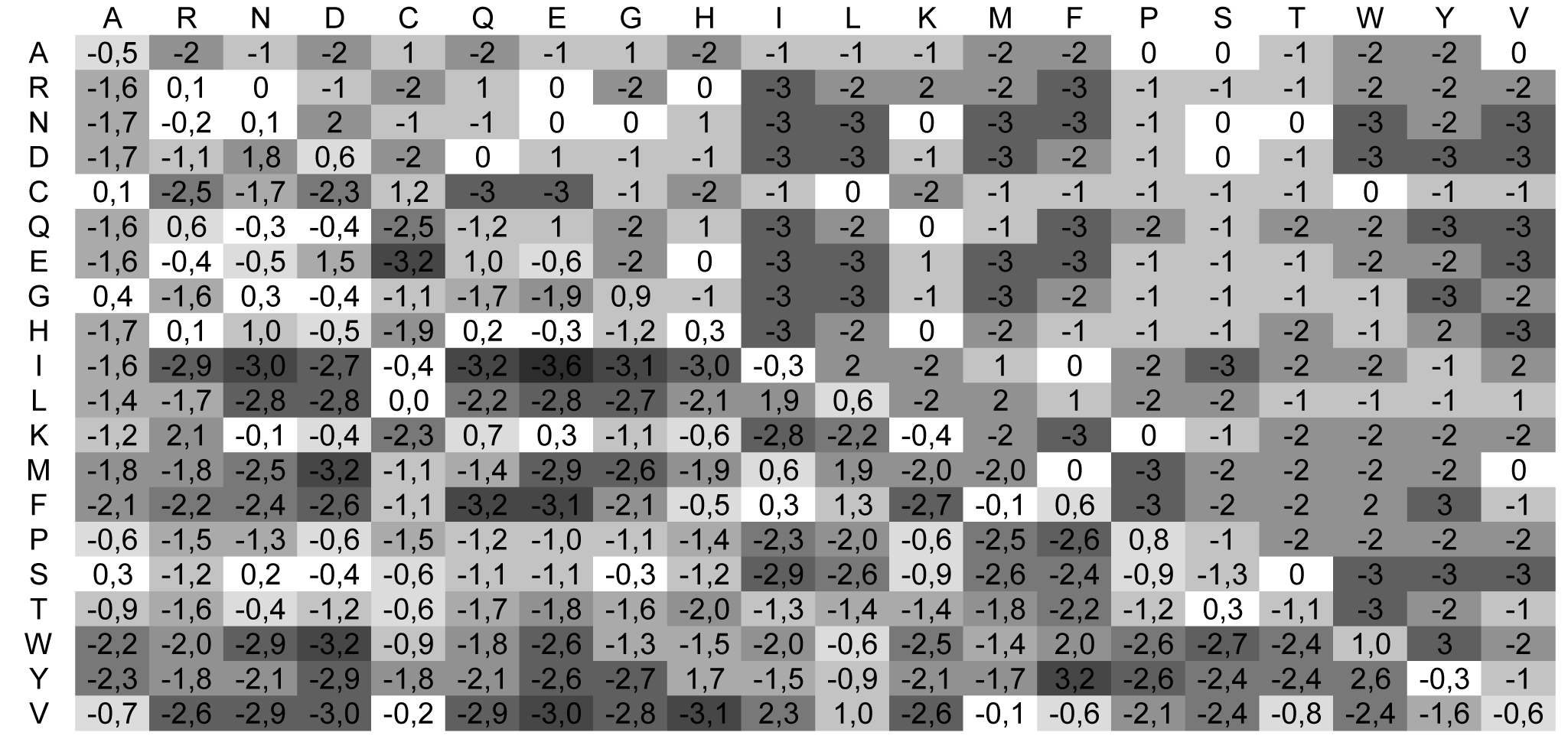

Supplement: Figure S8 — Reconstruction of the BLOSUM62 matrix – stage 0, G-matrix and no eigenvectors. The 6 stages of reconstruction of BLOSUM62 matrix with eigenvectors from BLOSUM100 are presented in Figures S8–S13. The stage 0 involves the non-specific G-matrix only, stage 1 involves the first eigenvector of BLOSUM100, and k-th stage involves k eigenvectors. For each matrix the triangle above diagonal displays differences between matrices scaled to half bit units and rounded, the diagonal and triangle below displays differences between matrices scaled to half bit units and not rounded. The shades of gray correspond to the differences. The gray scale is presented in Figure S14. (TIF) [file pone.0098983.s008.tif]

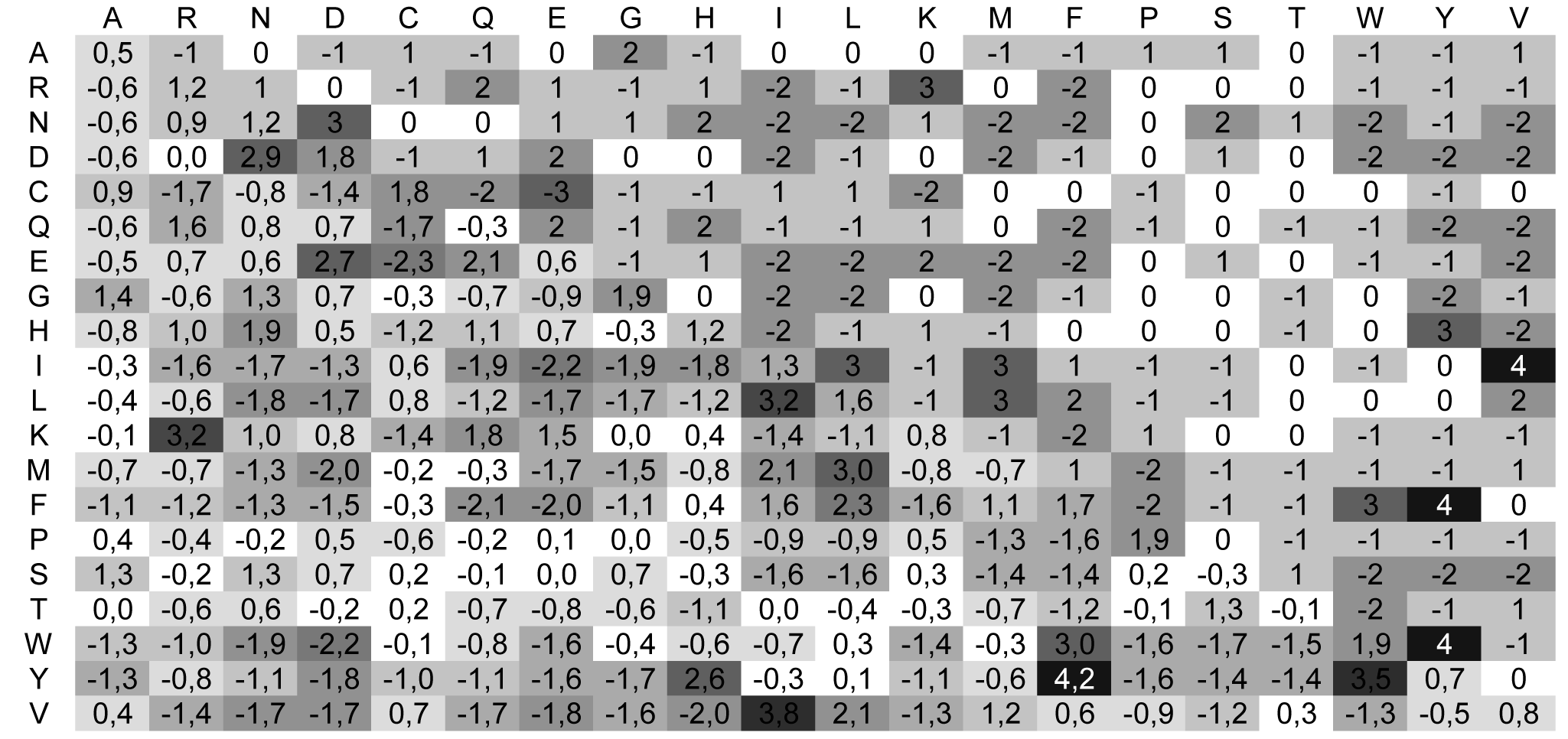

Supplement: Figure S9 — Reconstruction of the BLOSUM62 matrix – stage 1, G-matrix and one eigenvector. (TIF) [file pone.0098983.s009.tif]

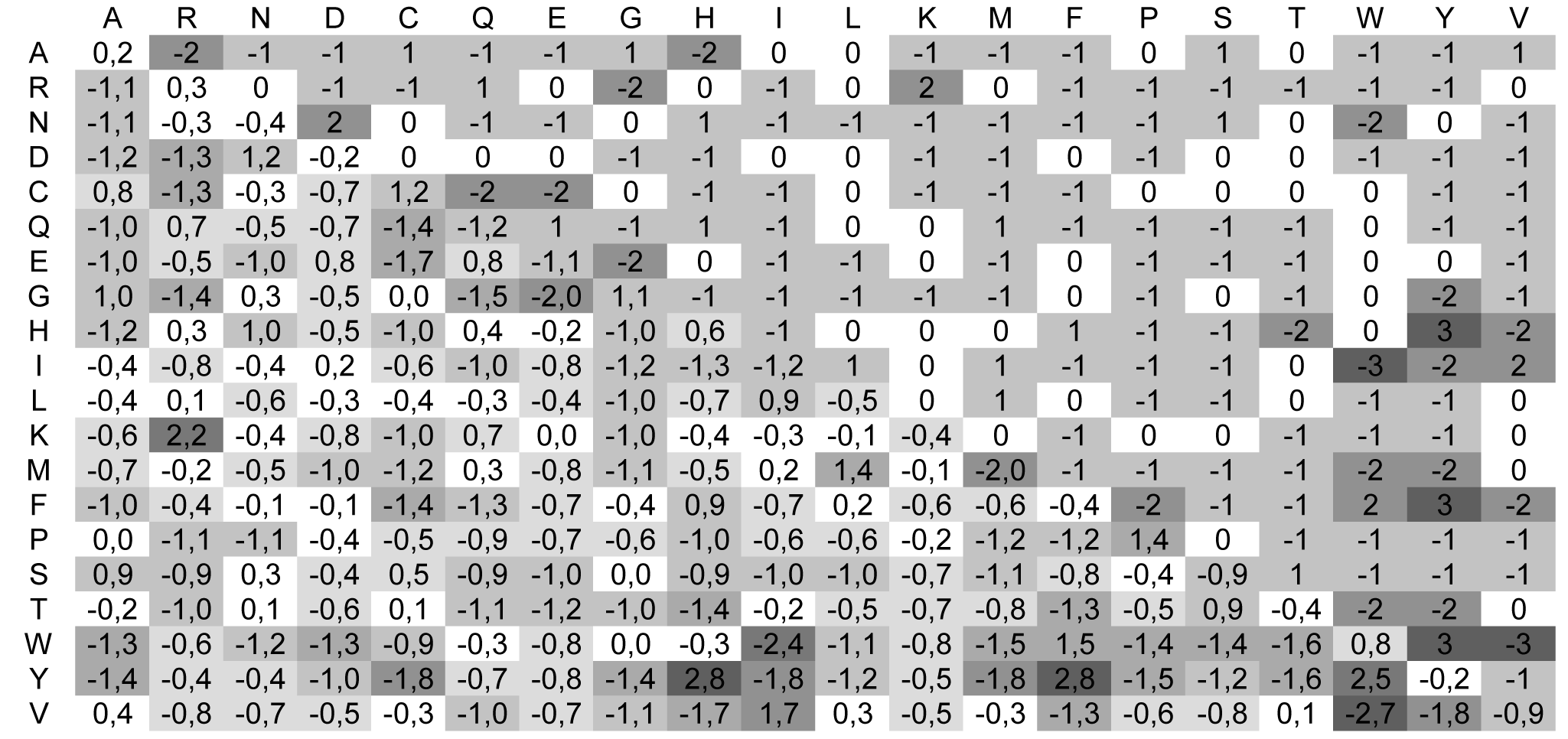

Supplement: Figure S10 — Reconstruction of the BLOSUM62 matrix – stage 2, G-matrix and two eigenvectors. (TIF) [file pone.0098983.s010.tif]

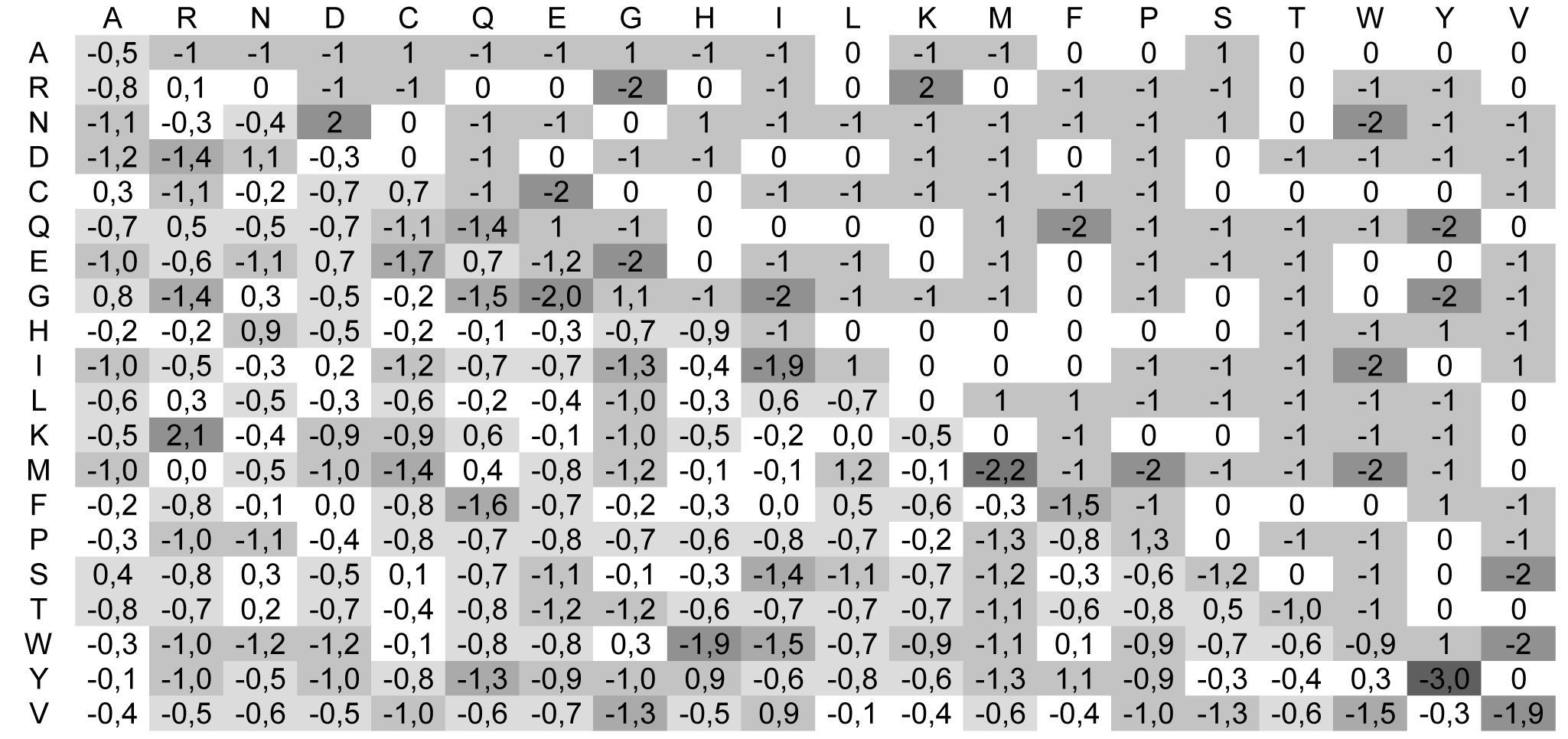

Supplement: Figure S11 — Reconstruction of the BLOSUM62 matrix – stage 3, G-matrix and three eigenvectors. (TIF) [file pone.0098983.s011.tif]

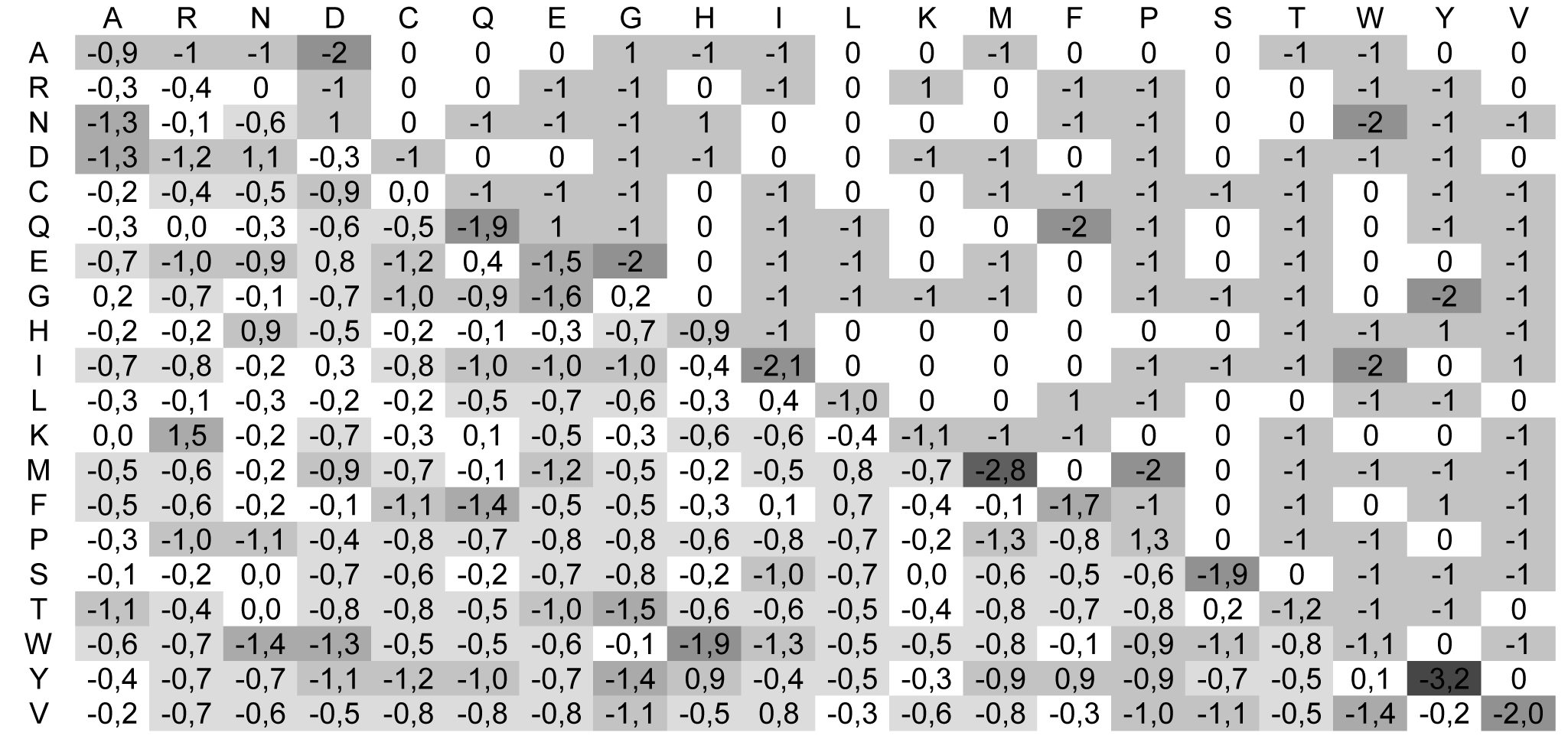

Supplement: Figure S12 — Reconstruction of the BLOSUM62 matrix – stage 4, G-matrix and four eigenvectors. (TIF) [file pone.0098983.s012.tif]

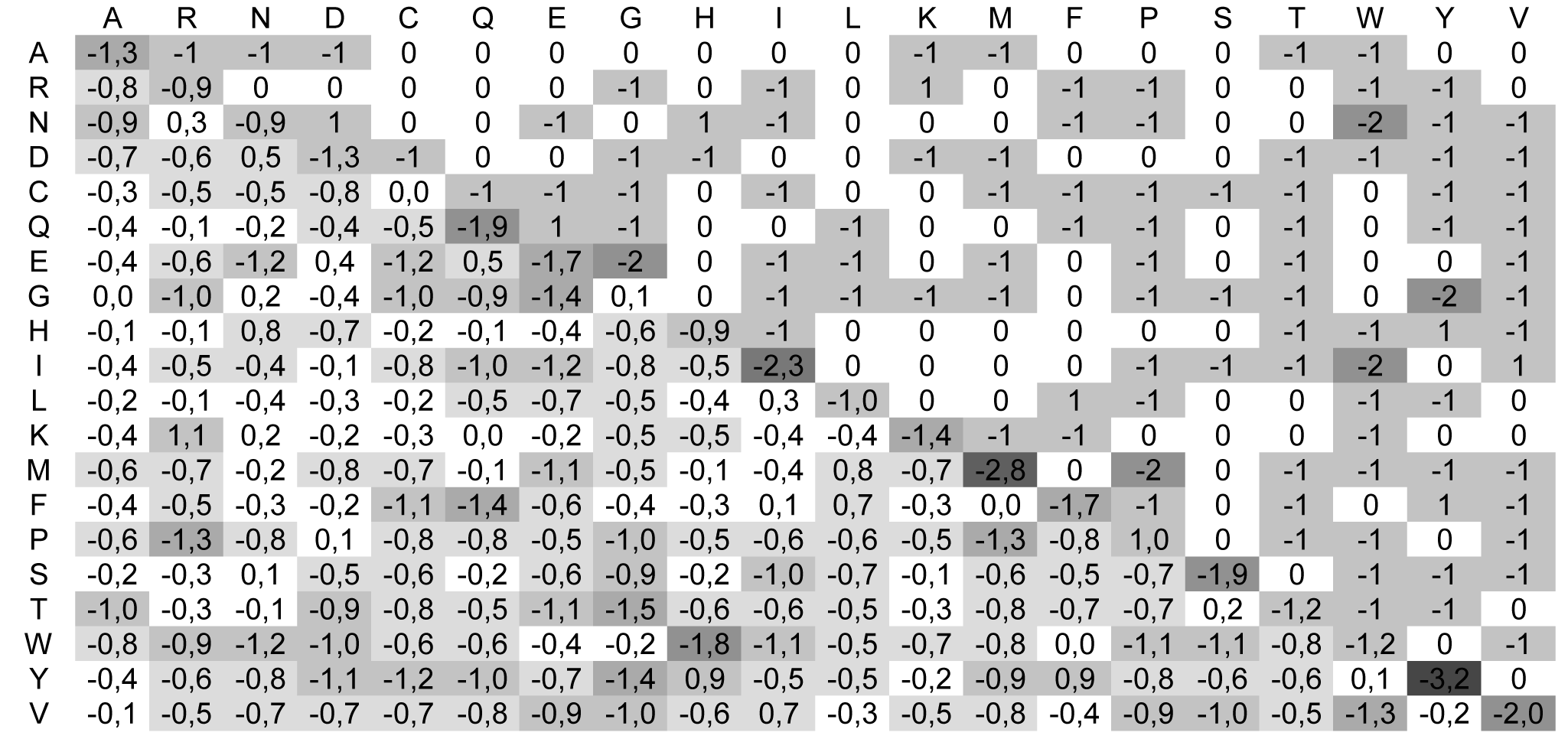

Supplement: Figure S13 — Reconstruction of the BLOSUM62 matrix – stage 5, G-matrix and five eigenvectors. (TIF) [file pone.0098983.s013.tif]

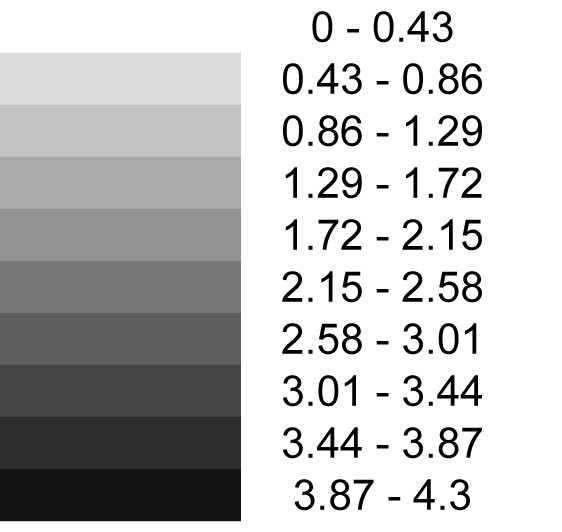

Supplement: Figure S14 — The scale for Figures S8–S13. The shades of gray correspond to the differences between original and reconstructed matrices. The maximal value for the scale is obtained as the absolute value of the off-diagonal elements of the original BLOSUM62 matrix, the minimal value is zero. The scale is divided equally into ten intervals. (TIF) [file pone.0098983.s014.tif]

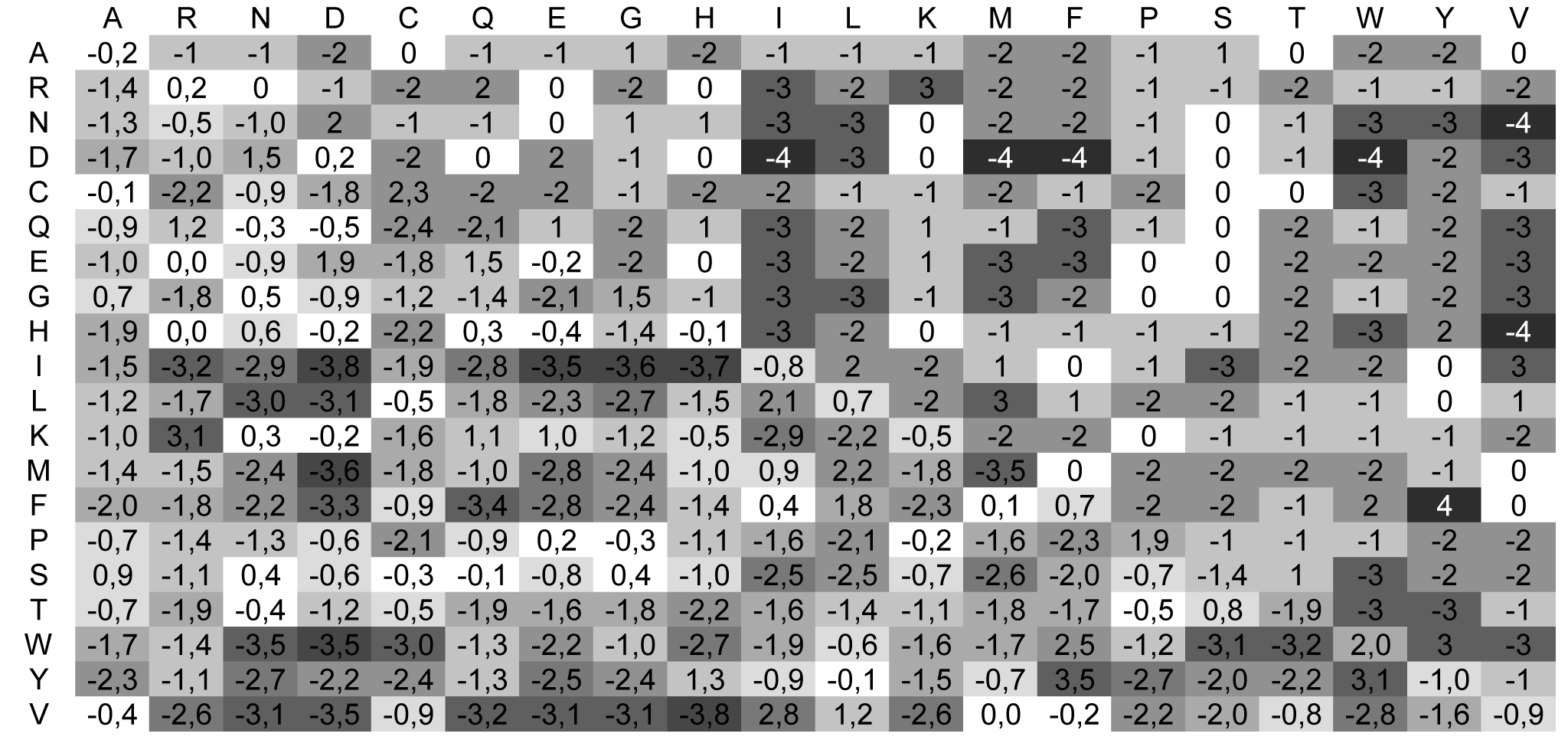

Supplement: Figure S15 — Reconstruction of the BLOSUM45 matrix – stage 0, G-matrix and no eigenvectors. The 5 stages of reconstruction of BLOSUM45 matrix with eigenvectors from BLOSUM100 are presented in Figures S15–S19. The stage 0 involves the non-specific G-matrix only, stage 1 involves the first eigenvector of BLOSUM100, and k-th stage involves k eigenvectors. For each matrix the triangle above diagonal displays differences between matrices scaled to 1/3 bit units and rounded, the diagonal and triangle below displays differences between matrices scaled to 1/3 bit units and not rounded. The shades of gray correspond to the differences. The gray scale is presented in Figure S20. (TIF) [file pone.0098983.s015.tif]

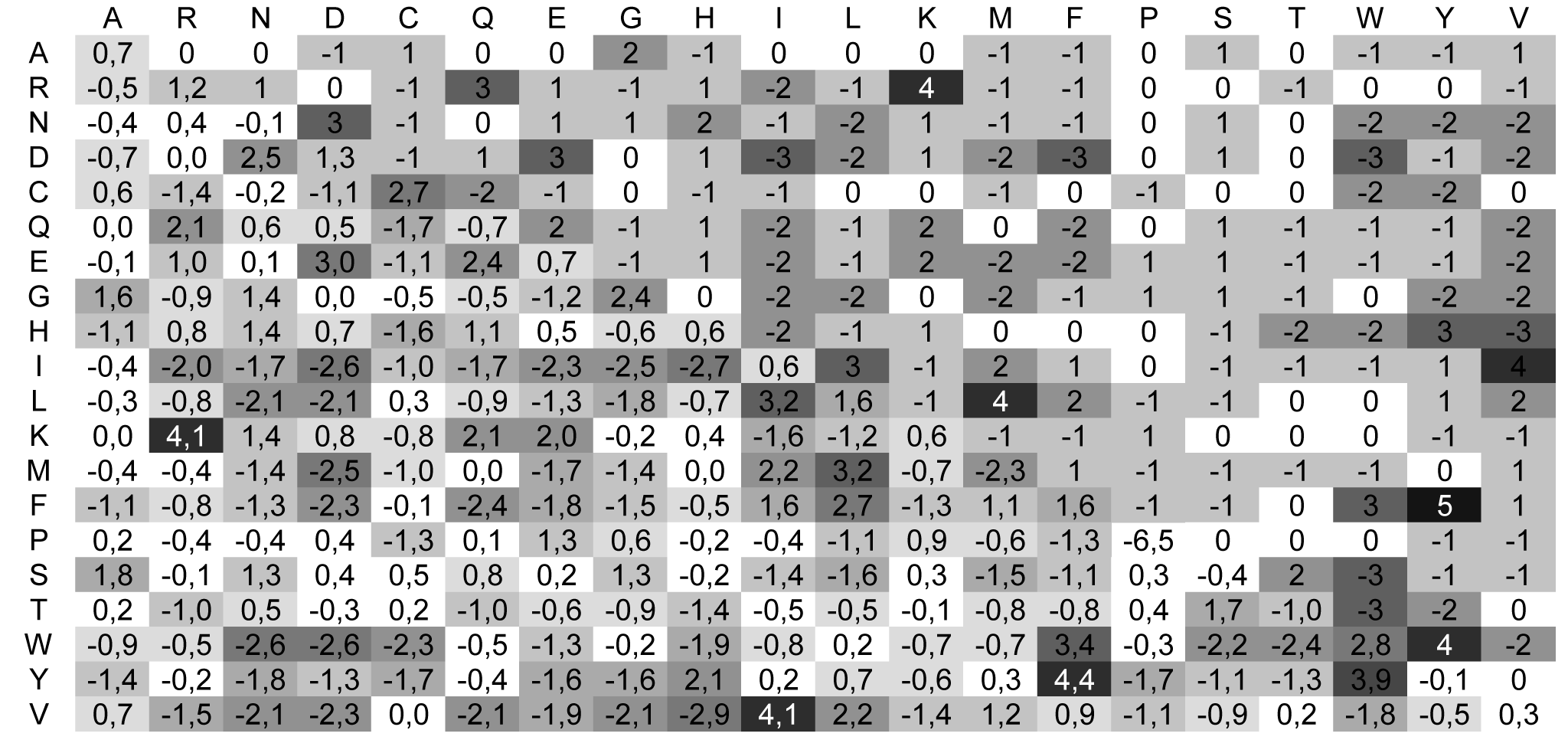

Supplement: Figure S16 — Reconstruction of the BLOSUM45 matrix – stage 1, G-matrix and one eigenvector. (TIF) [file pone.0098983.s016.tif]

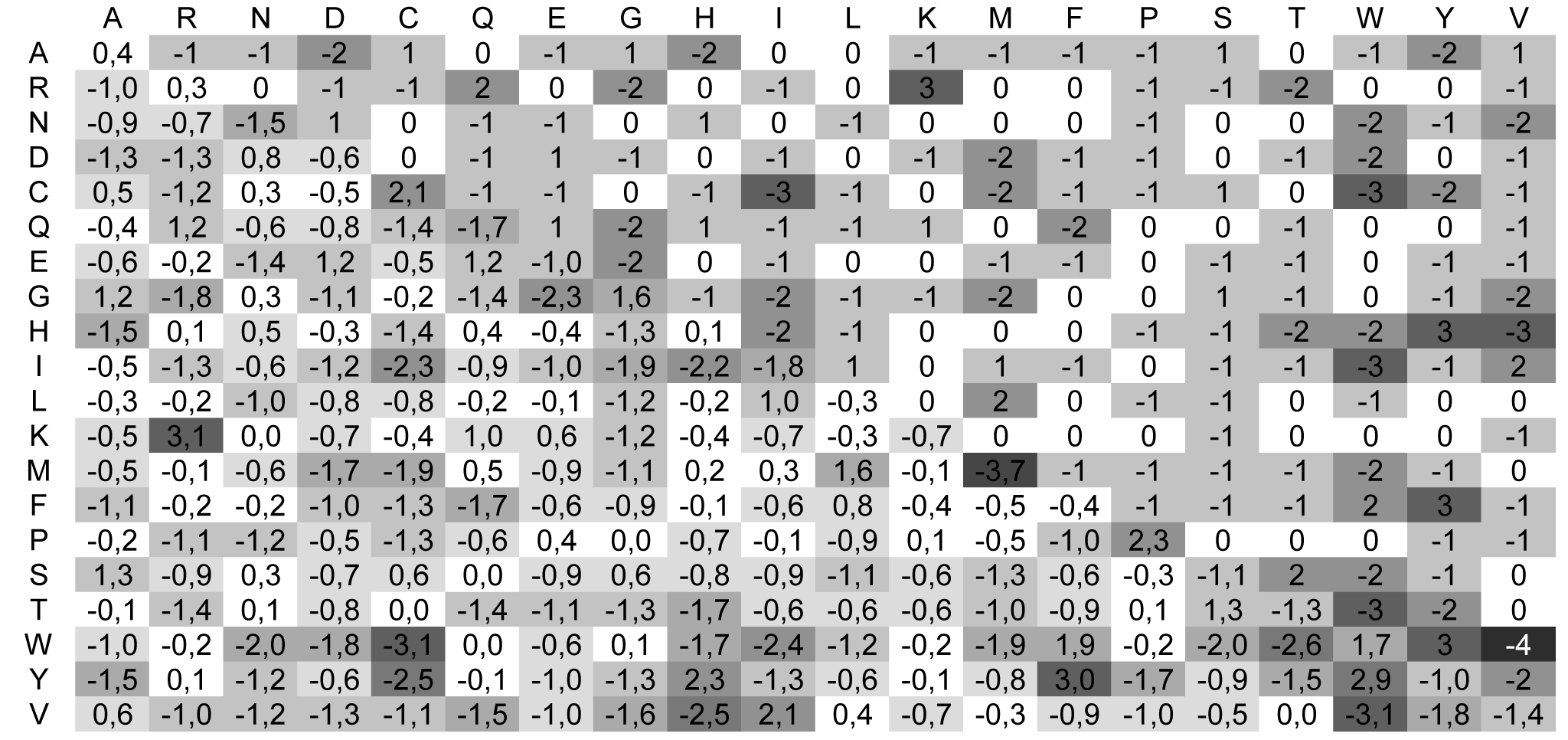

Supplement: Figure S17 — Reconstruction of the BLOSUM45 matrix – stage 2, G-matrix and two eigenvectors. (TIF) [file pone.0098983.s017.tif]

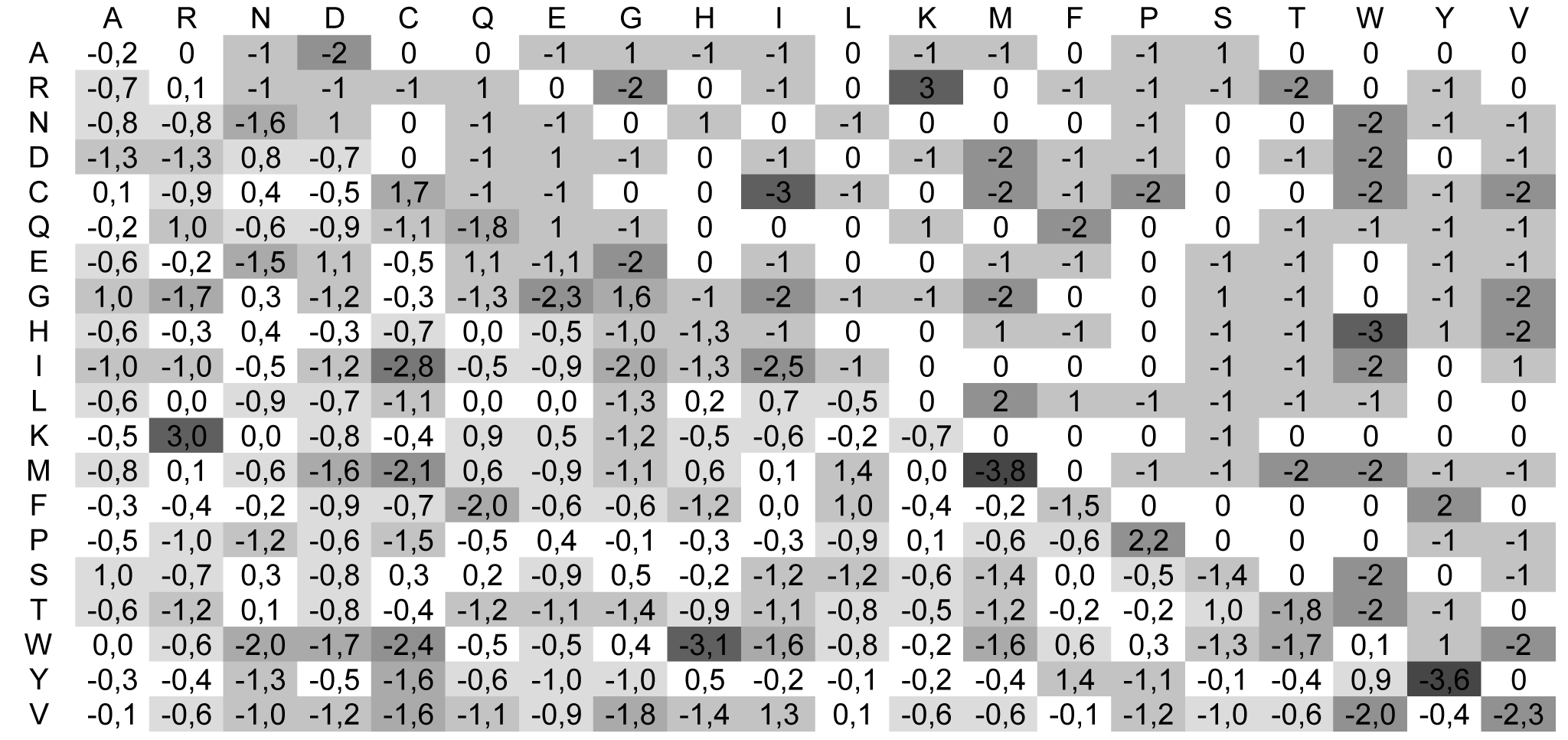

Supplement: Figure S18 — Reconstruction of the BLOSUM45 matrix – stage 3, G-matrix and three eigenvectors. (TIF) [file pone.0098983.s018.tif]

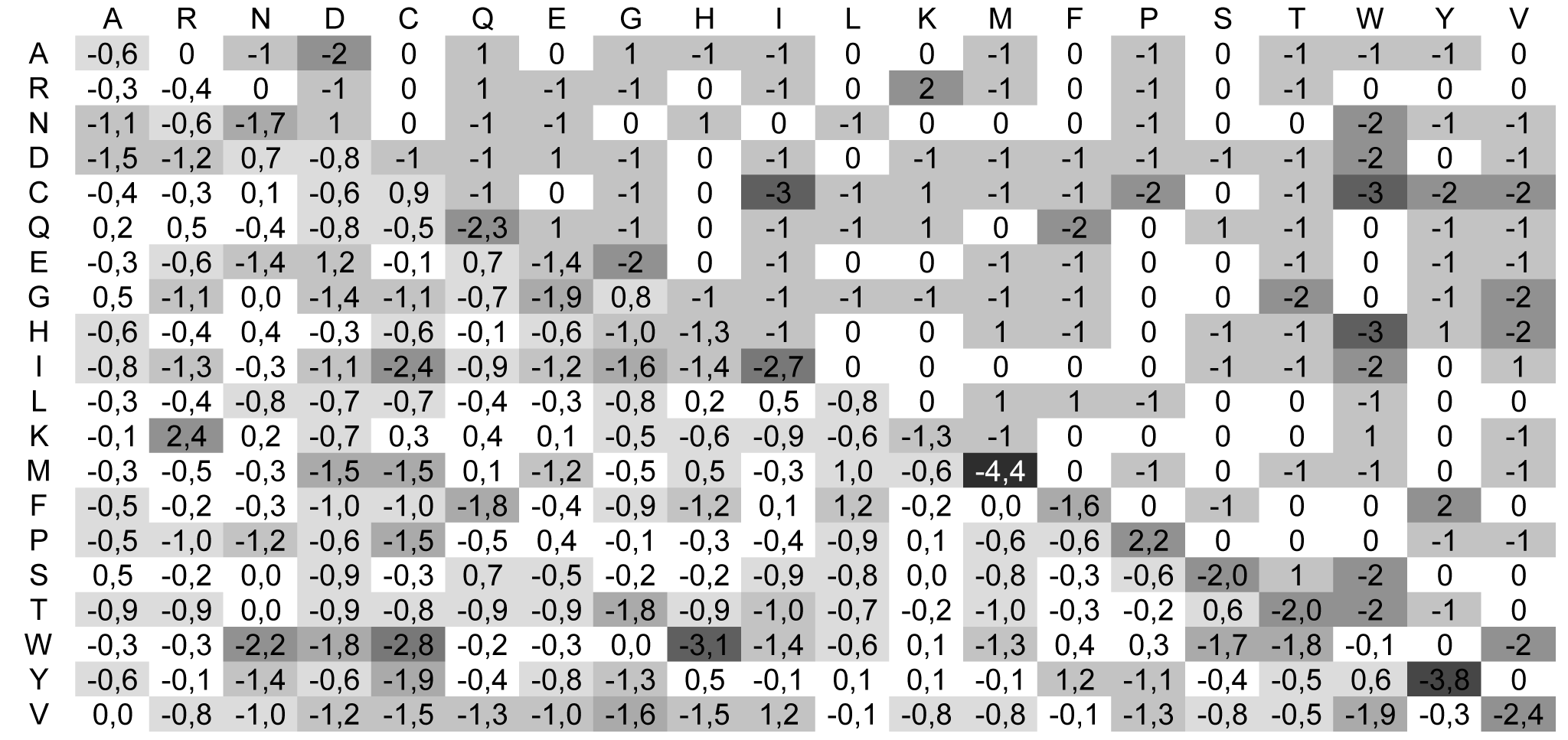

Supplement: Figure S19 — Reconstruction of the BLOSUM45 matrix – stage 4, G-matrix and four eigenvectors. (TIF) [file pone.0098983.s019.tif]

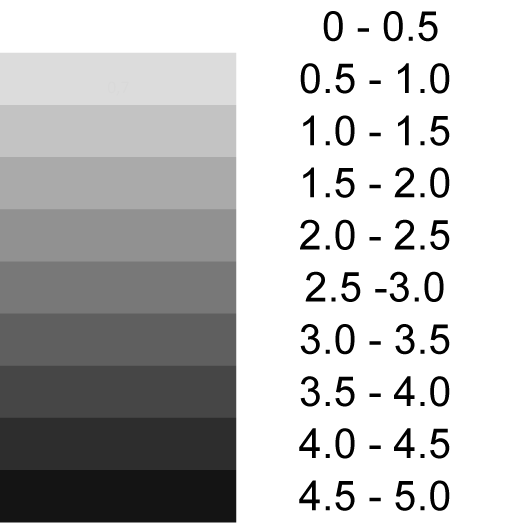

Supplement: Figure S20 — The scale for Figures S15–S19. The shades of gray correspond to the differences between original and reconstructed matrices. The maximal value for the scale is obtained as the absolute value of the off-diagonal elements of the original BLOSUM45 matrix, the minimal value is zero. The scale is divided equally into ten intervals. (TIF) [file pone.0098983.s020.tif]
